# MyomiR Networks in Spinal Muscular Atrophy: Associations With Clinical Severity and Treatment Response

**DOI:** 10.1007/s12035-026-05862-4

**Published:** 2026-04-30

**Authors:** Maruša Barbo, Blaž Koritnik, Lea Leonardis, Vita Dolžan, Metka Ravnik-Glavač

**Affiliations:** 1 Institute of Biochemistry and Molecular Genetics, Faculty of Medicine, University of Ljubljana, Ljubljana, Slovenia; 2 Institute of Clinical Neurophysiology, University Medical Centre Ljubljana, Ljubljana, Slovenia; 3 Department of Neurology, Faculty of Medicine, University of Ljubljana, Ljubljana, Slovenia

**Keywords:** Spinal muscular atrophy, Biomarkers, myomiR, lncRNA, *SMN2* splicing, miR-206

## Abstract

Disease-modifying therapies have significantly influenced the clinical course of spinal muscular atrophy (SMA), yet objective biomarkers for monitoring disease progression and treatment remain limited. We profiled four muscle-specific miRNAs (myomiRs), ten bioinformatically predicted mRNA targets, two functionally associated lncRNAs, and *SMN* transcripts in whole blood from 50 adults with SMA types II–IV. Using RT-qPCR, we assessed associations between baseline RNA expression and demographic and clinical parameters, including SMA type, ambulatory status, motor and respiratory function, and explored longitudinal changes during nusinersen (24 months) and risdiplam (6/12 months) treatment. At baseline, miR-206 was higher in type III than in type II and in ambulatory compared to non-ambulatory patients, while it correlated positively with motor and respiratory function and with *SMN* mRNA variants (total, FL, and ∆7). *SMN* transcript levels were higher in patients with more *SMN2* copies and in ambulatory patients and showed positive correlations with motor and respiratory function. miR-133a-3p and miR-133b correlated negatively with upper limb and respiratory function, and sex-related differences were observed for miR-133a-3p, *FGFR1*, *ANXA2*, and *LINCMD1*. During nusinersen treatment, we observed a decrease in miR-206, *LINCMD1*, and *lnc-GJA1-2*, alongside modest reductions in *SMN*-∆7 and total *SMN*. In contrast, risdiplam induced a peripheral splicing shift: *SMN*-FL and the FL/∆7 ratio increased, while *SMN*-∆7 decreased; miR-133a-3p also decreased at 6 months. By integrating muscle-derived RNAs, particularly miR-206, with blood *SMN2* splicing changes, we propose a composite, blood-based biomarker approach for assessing SMA status and treatment-associated molecular changes and highlight myomiR-lncRNA-mRNA networks that suggest disease-relevant mechanisms.

**Supplementary Information:**

The online version contains supplementary material available at 10.1007/s12035-026-05862-4.

## Introduction

Spinal muscular atrophy (SMA) is a genetic neuromuscular disorder characterized by progressive muscle weakness and wasting. The disease is caused by the degeneration of motor neurons (MNs). Based on the age of onset of symptoms and the severity of the disease, SMA can be categorized into four main clinical types, types I–IV [1]. A distinct and most severe form of the disease is SMA type 0 and is characterized by neonatal onset. It manifests as respiratory distress already at birth [2]. The symptoms of SMA type I, the most common severe form, typically begin at around 6 months of age. The disease is manifested by the inability to sit and a life expectancy of less than 2 years. In contrast, SMA type II, an intermediate type, occurs after the age of 6 months. SMA type II patients can sit unaided but never walk. SMA type III is a milder form that usually develops after the age of 18 months. Those affected can walk unaided but gradually lose this ability. Finally, SMA type IV affects adults, often in the second or third decade of life, and is characterized by a moderate clinical course [1].

The onset of SMA is associated with mutations in both alleles of the telomeric survival of motor neuron 1 (*SMN1*) gene, which results in the deficiency of the survival of motor neuron (SMN) protein [3]. However, the severity of the disease varies among SMA patients and is influenced by variations in the copy number of the paralogous centromeric survival of motor neuron 2 (*SMN2*) gene [4–6]. Lacking exon 7, the *SMN2* gene produces a protein with reduced self-oligomerization and stability [7, 8]. While the severity of SMA is known to be majorly impacted by the number of *SMN2* copies, this association is not fully predictive. There are reports of siblings with the same *SMN1* mutations and the same *SMN2* copy numbers, but with markedly different disease manifestations [9, 10].

Currently, three therapies aimed at increasing SMN protein production have been approved by both the U.S. Food and Drug Administration (FDA) and the European Medicines Agency (EMA) [11]. Onasemnogene abeparvovec (Zolgensma; Novartis) is a viral-vector based gene therapy approved for patients with 5q-associated SMA resulting from bi-allelic *SMN1* mutations, who have a clinical diagnosis of SMA type I or carry up to three *SMN2* copies [12]. Nusinersen (Spinraza; Biogen), an antisense oligonucleotide, and risdiplam (Evrysdi; Roche), a small molecule *SMN2* pre-mRNA splicing modifier, are both designed to increase the inclusion of exon 7 and thus increase the production of functional SMN protein. Despite the shared mechanism, they differ in both their therapeutic modality and route of administration: nusinersen is delivered intrathecally, whereas risdiplam is administered orally [13].

The advent of SMN-restorative therapies has significantly improved the disease course for patients with SMA. However, these therapies have also led to the emergence of new disease phenotypes, making it increasingly difficult to establish reliable measures for evaluating treatment efficacy [14, 15]. Treatment outcomes depend strongly on early intervention, which is crucial to fully address SMN-related deficits [16, 17]. As gene therapy is largely limited to infants and very young children, patients with disease onset later in childhood or adulthood, who constitute an estimated two-thirds of all SMA patients, are treated with *SMN2* splicing modifiers [18]. However, nusinersen, the first approved and most widely used drug, shows a variable clinical response in patients with SMA types II and III, with 30–40% of patients achieving clinically meaningful improvement [19, 20]. The high clinical variability observed in SMA also highlights the possible role of additional modifying factors besides *SMN2* copy number influencing disease severity, progression and treatment response, with epigenetic mechanisms being plausible candidates.

Epigenetic regulation plays an essential role in maintaining the functionality of the genome, as it serves as the main mechanism for regulating gene expression. The epigenomic landscape is orchestrated by various mechanisms, including DNA methylation, histone modifications, chromatin remodeling, and non-coding RNAs (ncRNAs) [21]. MicroRNAs (miRNAs) are short regulatory ncRNAs with a length of around 22 bp that are being extensively researched for their influence on post-transcriptional gene regulation [22]. They simultaneously regulate the expression of multiple target genes by binding to their target mRNAs, thereby influencing the activity of various associated signaling pathways. MiRNAs are involved in the development, function, and survival of spinal MNs, particularly by modulating cytoskeletal structure, synapse formation, and axonal growth [23–27]. It has been demonstrated that dysregulation or aberrant expression of miRNAs plays a major role in the pathophysiology of several neurodegenerative diseases [28, 29], including SMA [26, 30]. To elaborate, the selective susceptibility of MNs appears to stem from various mechanisms, potentially including alterations in the expression of MN-specific miRNAs [26]. In addition, the SMN protein interacts directly with proteins involved in miRNA synthesis and function and is therefore likely involved in miRNA biogenesis [31–33]. SMN deficiency, a hallmark of SMA, affects not only miRNAs but also multiple target mRNAs in the MNs simultaneously [34], providing a plausible explanation for why SMN depletion affects so many pathways [26].

Researchers are continuously searching for reliable biomarkers to improve patient classification, follow disease progression, and assess response to treatment, preferably using minimally invasive methods [23, 29, 35]. Among the most promising candidates are miRNAs, which are secreted by cells either actively or in response to external stimuli, leading to measurable changes in their biofluid levels [36]. In addition to their involvement in the survival and function of spinal MNs, miRNAs have also been identified in neuromuscular junctions and muscles. To elaborate, muscle-specific miRNAs (myomiRs) are a group of miRNAs expressed in muscle, including miR-1, miR-133a, miR-133b, and miR-206, which play a crucial role in the regulation of myogenesis, muscle degeneration, and the processes of proliferation, differentiation, and regeneration of skeletal muscle [37]. Therefore, changes in the levels of specific miRNAs in human biofluids could serve as useful biomarkers for tracking SMA progression and evaluating the impact of therapeutic interventions. In a previous review, we summarized published data on miRNAs implicated in SMA pathogenesis that could potentially serve as biomarkers, and highlighted the most frequently dysregulated miRNAs associated with SMA, including the myomiRs mentioned above [38]. We focused on miR-133a/miR-133b and miR-1/miR-206 families, located on three different chromosomes [39], which are among the best-studied myomiRs. Briefly, Malacarne et al. reported increased serum miR-206 levels in SMA type II and type III patients compared with healthy controls [40], whereas Catapano et al. found no significant difference in miR-206 levels and no correlation with motor performance [41]. Bonanno et al. further showed that nusinersen treatment was associated with a significant decrease in miR-133a, miR-133b, and miR-1 levels, with miR-206 showing a similar downward trend, and the reduction in miR-133a also correlated with functional improvement [42]. In CSF, Magen et al. demonstrated that lower baseline levels of miR-133a-3p and miR-206 predicted a better clinical outcome following nusinersen treatment, while miR-206 levels were inversely associated with functional scores [43].

In addition to exploring several miRNAs and their targets in the context of SMA, we also focused on long non-coding RNAs (lncRNAs), another subset of ncRNAs that are over 200 nucleotides in length. Compared with small ncRNAs, lncRNAs remain less well characterized, yet they appear to play important regulatory roles in the human genome. Various functions are attributed to lncRNAs, including the modulation of miRNA activity [44], regulation of cellular differentiation and homeostasis [45], and stemness maintenance [46]. As they are also strongly expressed in the central nervous system (CNS), it is not surprising that their dysregulation has been linked to the pathogenesis of neurodegenerative diseases [47, 48].

This study presents a targeted analysis of the expression profiles of selected RNAs, including miRNAs, their predicted mRNA targets and linked lncRNAs in peripheral whole blood samples from patients with SMA. Our aim was to assess the potential of these ncRNAs as molecular biomarkers for monitoring disease progression and response to treatment, while also providing insight into the regulatory networks underlying SMA pathophysiology. We compared baseline RNA expression before treatment initiation with respect to demographic and clinical parameters and investigated longitudinal changes in RNA expression in patients receiving SMN restorative therapies to assess treatment-associated dynamics. Finally, we examined the expression of disease-associated *SMN* transcript variants to evaluate their potential clinical significance and provide biological context for interpreting the expression patterns of selected RNAs and their treatment-related dynamics.

## Materials and Methods

### Study Subjects and Samples

A total of 50 adult patients with SMA, treatment-naïve at baseline, were included in the study. Patients were routinely followed at the Department of Neurology, University Medical Centre Ljubljana, Slovenia, from September 2019 to January 2024. The inclusion criteria for the study were a genetically and clinically confirmed diagnosis of SMA, ongoing treatment with nusinersen or risdiplam, and the availability of comprehensive demographic and clinical data. Blood samples from the nusinersen-treated cohort were collected at baseline (T0, before the initial dose) and after 24 ± 6 months (T24), while samples from patients receiving risdiplam were collected at baseline (T0), and after 6 ± 3 months (T6) and 12 ± 3 months (T12). Data were collected on sex, SMA type, *SMN1* gene mutation, *SMN2* copy number, age at first blood sampling, and ambulatory status. Genetic data, including *SMN1* genotype and *SMN2* copy number when available, were collected retrospectively from clinical records generated during routine diagnostic evaluation prior to enrolment in the present study.

The functional assessments encompassed the evaluation of the patients' motor functions using the Revised Hammersmith Scale (RHS) and the Revised Upper Limb Module (RULM) as well as pulmonary function tests, including vital capacity percent predicted (VC%) and peak expiratory flow percent predicted (PEF%). All assessments were performed by qualified clinical assessors following the center’s standard clinical practices. Statistical analyses included clinical data from assessments conducted near the corresponding blood collection time points to ensure temporal alignment with the transcriptomic data. The interval between the reported age at onset of symptoms and the age at blood collection at baseline was used to calculate the disease duration.

Approval for the study was provided by the National Medical Ethics Committee of the Republic of Slovenia (0120–293/2019/8, 120–26/2024–2711-3), and the study was carried out according to the Declaration of Helsinki. Each participant signed an informed consent.

### Study Design and Selection of Candidate RNAs

The study was performed in three consecutive stages, the first of which aimed to identify the candidate miRNAs implicated in SMA through a literature review of publicly available human studies on circulating miRNAs detected in blood, serum, plasma, or CSF of SMA patients to compile a list of the most frequently deregulated miRNAs between SMA patients and controls, as well as pre- and post-treatment in SMA patients [38].

Subsequently, we aimed to explore the regulatory interactions of selected miRNAs with their respective lncRNA interactors and potential mRNA targets. In brief, the March 2023 offline version of the RAID v2 database [49] was used to obtain miRNA interaction data. Interaction data were filtered and obtained automatically in the R 4.0.2 environment (R Core Team 2020, Vienna, Austria). Interaction networks illustrating the regulatory relationships among miRNAs, mRNAs and lncRNAs were visualized and pruned using Cytoscape (v3.8.2., Cytoscape Team) [50]. The resulting miRNA–lncRNA–mRNA axes were then analyzed focusing on key interaction hubs and clusters of genes regulated by multiple miRNAs. The gene ontology (GO) analysis for the identified clusters was also performed using the software package Cytoscape (v3.8.2., Cytoscape Team) with the integrated application ClueGO (v2.5.8, Laboratory of Integrative Cancer Immunology (Team 15), Paris, France) [51].

In the final stage of the study, we used the quantitative real-time polymerase chain reaction (qPCR) approach to evaluate the transcriptomic profiles, as detailed in the subsequent paragraphs.

### RNA Isolation

Whole blood samples were collected from patients with SMA into commercially available Tempus RNA blood tubes (Thermo Fisher Scientific, Waltham, MA, USA) for total RNA extraction. Total RNA was extracted using MagMAX™-96 Total RNA Isolation Kit (Thermo Fisher Scientific, Waltham, MA, USA) on the KingFisher Duo Prime sample preparation instrument (Thermo Fisher Scientific, Waltham, MA, USA) as specified in the manufacturer’s protocol. The quantity and purity of total RNA were determined spectrophotometrically using Lambda Bio (PerkinElmer, Waltham, MA, USA).

### Reverse Transcription

To analyze miRNA expression, total RNA was diluted to a maximum concentration of 50 ng/µl. Total isolated miRNA was reverse transcribed using the TaqMan Advanced miRNA cDNA Synthesis Kit (Applied Biosystems, Waltham, MA, USA). cDNA synthesis was performed according to the manufacturer’s workflow, including poly(A) tailing, adaptor ligation, reverse transcription, and miR-Amp preamplification. For each reaction, 2 µl of total RNA was first subjected to poly(A) tailing in a 5 µl reaction containing 3 µl Poly(A) Reaction Mix, followed by incubation at 37 °C for 45 min and 65 °C for 10 min. Adaptor ligation was then performed by adding 10 µl Ligation Reaction Mix to the poly(A) tailing product, followed by incubation at 16 °C for 60 min. Reverse transcription was performed by adding 15 µl RT Reaction Mix to the ligation product, followed by incubation at 42 °C for 15 min and 85 °C for 5 min. The resulting cDNA was then subjected to miR-Amp preamplification in a 50 µl reaction containing 5 µl RT product and 45 µl miR-Amp Reaction Mix, using the following cycling conditions: 95 °C for 5 min, followed by 14 cycles of 95 °C for 3 s and 60 °C for 30 s, then 99 °C for 10 min. The resulting cDNA was diluted 1:10 prior to PCR.

To evaluate the expression of target mRNAs, lncRNAs, and *SMN* transcript variants (*SMN*-FL and *SMN*-∆7), and total *SMN* transcripts (*SMN*-total), reverse transcription was performed using the High-Capacity cDNA Reverse Transcription Kit with RNase inhibitor (Applied Biosystems, Waltham, MA, USA), according to the manufacturer's instructions. For each reaction, 600 ng of total RNA (10 µl at 60 ng/µl) was reverse transcribed in a total reaction volume of 20 µL containing 2.0 µL 10 × RT buffer, 0.8 µL 25 × dNTP mix (100 mM), 2.0 µL RT random primers, 1.0 µL MultiScribe reverse transcriptase, 1.0 µL RNase inhibitor, and 3.2 µL RNase-free water. Reverse transcription was performed under the following conditions: 25 °C for 10 min, 37 °C for 120 min, and 85 °C for 5 min. The resulting cDNA was diluted 1:5 for mRNA target analysis and 1:10 for *SMN* transcript analysis prior to PCR.

For the analysis of lncRNA expression, cDNA was pre-amplified after reverse transcription using the TaqMan PreAmp Master Mix (Applied Biosystems, Waltham, MA, USA). Each pre-amplification reaction was prepared in a final volume of 7.5 µL, containing 3.75 µL TaqMan PreAmp Master Mix, 1.875 µL pooled TaqMan Gene Expression Assays, and 1.875 µL cDNA. Pre-amplification was performed for 10 cycles under the following conditions: 95 °C for 10 min, followed by 10 cycles of 95 °C for 15 s and 60 °C for 4 min, and a final step at 99 °C for 10 min. The pre-amplified cDNA was then diluted 1:5 prior to PCR. Subsequent relative expression analysis was performed on pre-amplified cDNA for both lncRNAs and endogenous controls to ensure comparability of threshold cycle (Ct) values. The cDNA samples were stored at −20 °C.

### Relative Expression Analysis

Detection and quantification of four miRNAs (miR-1-3p, miR-133a-3p, miR-133b, and miR-206), ten mRNAs (*PGD*, *G6PD*, *TKT*, *HDAC4*, *FGFR1*, *SP1*, *TGFB1*, *KCNQ1*, *IGF1R*, and *ANXA2*), and total *SMN* transcript levels (*SMN-*total) were performed using predesigned TaqMan Advanced MicroRNA Assays (Applied Biosystems, Waltham, MA, USA) and TaqMan Gene Expression Assays (Applied Biosystems, Waltham, MA, USA), respectively. The corresponding Assay IDs for all predesigned commercial assays are listed in Table S1.

Custom TaqMan Gene Expression Assays (Applied Biosystems, Waltham, MA, USA) were used to evaluate the expression of selected lncRNAs (*lnc-GJA1-2*, *LINCMD1*) and *SMN* transcript variants, including full-length *SMN* (*SMN*-FL) and the *SMN* transcript without exon 7 (*SMN*-∆7). For each transcript, specific primers and probes were designed as follows: for *lnc-GJA1-2*, the forward primer was 5'-GCTGAAGGGCTCGTCAAGT-3', the reverse primer 5'-CTCGGCACCTCCTCTGC-3', and the probe 5'-FAM-CTGGGCTCCCACTTTG-NFQ-3'. For *LINCMD1*, the forward primer was 5'-GAAGAAGAAACTCCCCAGAAAGGT-3', the reverse primer 5'-AGCTCTTTTCCCACCTGCTC-3', and the probe 5'-FAM-CCAT ACATCGTGAAGACTG-NFQ-3'. For *SMN*-FL, the forward primer was 5'-CATGAGTGGCTATCATACTGGCTATT-3', the reverse primer 5'-GAATGTGAGCACCTTCCTTCTTTTT-3', and the probe 5'-FAM-ATATGGGTTTTAGACAAAATC-NFQ-3'. For *SMN*-∆7, the forward primer was 5'-CATGGTACATGAGTGGCTATCATACTG-3', the reverse primer 5'-GTCTG ATCGTTTCTTTAGTGGTGTCA-3', and the probe 5'-FAM-ATGCCAGCATTTCCATATAA-NFQ-3'.

For miRNA expression analysis, PCR reactions were performed using TaqMan Fast Advanced Master Mix (Applied Biosystems, Waltham, MA, USA) in a final volume of 10 µL, containing 5.0 µL master mix, 0.5 µL TaqMan Advanced miRNA Assay, 2 µL RNase-free water, and 2.5 µL cDNA.

For expression analyses of mRNA targets, lncRNAs, *SMN* transcript variants (*SMN*-FL and *SMN*-∆7), and total *SMN* transcripts (*SMN*-total), PCR reactions were performed using TaqMan Fast Advanced Master Mix (Applied Biosystems, Waltham, MA, USA) in a final volume of 10 µL, containing 5.0 µL master mix, 0.5 µL predesigned or custom TaqMan Gene Expression Assay, 3.5 µL RNase-free water, and 1.0 µL cDNA.

All PCR reactions were performed in technical triplicates and run on the QuantStudio™ 7 Flex Real-Time PCR System (Applied Biosystems, Waltham, MA, USA) under the following thermal cycling conditions: 20 s at 95 °C, followed by 40 cycles of 1 s at 95 °C and 20 s at 60 °C. For downstream analyses, only samples with a final standard deviation (SD) of Ct values below 0.5 were retained. Samples without a measurable amplification cycle by cycle 40 were classified as undetermined and assigned a Ct value of 40. According to this criterion, all analyzed miRNAs, mRNA targets, lncRNAs, *SMN* transcript variants, and total *SMN* transcripts were detected in each sample, except for miR-206, which was detected in 84 out of 96 samples (87.5%), and *LINCMD1*, which was detected in 81 out of 94 samples (86.2%). The analysis was conducted using QuantStudio Software v1.7.2 (Applied Biosystems, Waltham, MA, USA).

For normalization of miRNA expression, miR-191-5p and miR-16-5p were selected as endogenous controls. miR-16-5p was chosen based on its previous use in SMA serum myomiR studies and earlier serum RT-qPCR normalization studies [40, 42, 52], while miR-191-5p was included as an additional literature-based stable circulating reference miRNA [53]. In our cohort, both miRNAs were stably expressed across SMA samples, as indicated by SD of Ct values below 0.3. *ACTB* and *RPLP0* were used as endogenous control genes to normalize the expression of target mRNAs, lncRNAs, *SMN* transcript variants, and total *SMN* transcripts. The Ct values of selected candidate RNAs were normalized to the geometric mean of the Ct values of the corresponding control RNAs, and the relative RNA expression was calculated using the 2^−ΔCt^ method. Statistical analyses were performed on log-transformed expression data to reduce the effects of skewed data. Longitudinal changes were evaluated using the 2^−ΔΔCt^ method, with the pre-treatment time point (T0) serving as the calibrator for ΔΔCt calculations.

### Statistical Analysis

Descriptive statistics were applied to summarize the characteristics of patients with SMA. The normality of the data was tested using the Shapiro–Wilk test. Categorical variables were presented as frequencies (percentages), while continuous variables were presented as means with SD for normally distributed data or as medians with interquartile range (IQR) for non-normally distributed data.

The Mann–Whitney U test was used to assess significance between two groups of continuous variables with non-normal distributions, while the Student’s *t*-test was used for normally distributed variables. ANCOVA was performed to adjust for covariates, including age at blood collection, disease duration, and *SMN2* copy number. To evaluate the correlations between continuous variables, the Pearson correlation coefficient was applied for normally distributed variables and the Spearman’s rank correlation coefficient for non-normally distributed variables. For covariate-adjusted analyses, partial Pearson correlation coefficients were estimated, adjusting for age, disease duration, and *SMN2* copy number.

To evaluate longitudinal changes in RNA expression, only subjects with data from at least two time points were included in the final dataset. Longitudinal data were analyzed using a one-sample *t*-test for normally distributed variables and a Wilcoxon signed-rank test for non-normally distributed variables. Values of *p* less than 0.05 were considered statistically significant. All statistical analyses were performed using two-tailed tests. Data were analyzed using IBM SPSS Statistics (v31.0.1.0; IBM Corp., Armonk, NY, USA) and GraphPad Prism (v10.6.1; GraphPad Software, San Diego, CA, USA) was used for graph generation.

## Results

### Study Population

A total of 50 patients with SMA were included in the study. The demographic and clinical characteristics of patients with SMA are shown in Table 1. The study comprised 27 (54.0%) female participants and 23 (46.0%) male participants. 22 (45.8%) patients had SMA type II, and 23 (47.9%) had SMA type III, while only three patients (6.3%) had SMA type IV. Genetic data collected retrospectively from clinical records showed that 49 of 50 patients carried a *SMN1* deletion consistent with 5q-SMA, whereas one patient carried a compound heterozygous *SMN1* genotype, consisting of an *SMN1* deletion on one allele and a pathogenic *SMN1* point mutation on the other. Retrospectively collected *SMN2* copy number data were available for 45 patients: one (2.2%) SMA patient had two *SMN2* copies, 24 (53.3%) patients had three *SMN2* copies, 18 (40.0%) had four *SMN2* copies, and two (4.4%) patients had five *SMN2* copies. *SMN2* copy number was strongly associated with SMA type (*p* < 0.001). The median age (25th–75th percentile) at first blood sampling (baseline, T0) was 42.7 (30.8–52.1) years.

**Table 1:** Demographic and clinical characteristics of SMA patients (n = 50)

Characteristic	Category/Unit	SMA patients	SMA type II (n = 22)	SMA type III (n = 23)	*p*-value
Sex	Male, n (%)	23 (46.0)	8 (36.4)	13 (56.5)	0.236
	Female, n (%)	27 (54.0)	14 (63.3)	10 (43.5)	
SMA type	II, n (%)	22 (45.8) #2	/	/	/
	III, n (%)	23 (47.9)			
	IV, n (%)	3 (6.3)			
Number of *SMN2* copies	2, n (%)	1 (2.2) #5	0 #3	0 #2	**0.002**
	3, n (%)	24 (53.3)	16 (84.6)	7 (33.3)	
	4, n (%)	18 (40.0)	3 (15.8)	13 (61.9)	
	5, n (%)	2 (4.4)	0	1 (4.8)	
Age at blood collection (T0)	Years, median (25–75%)	42.7 (30.8–52.1)	31.2 (26.2–72.3)	49.9 (42.4–60.5)	** < 0.001**
Disease duration (T0)	Years, median (25–75%)	34.0 (25.0–45.0)	29.0 (23.5–40.5)	39.5 (32.5–46.8)	**0.046**
RHS (T0)	n, median (25–75%)	4.00 (1.50–16.0) #1	2.00 (0–4.00)	8.00 (2.00–31.0)	**0.003**
RULM (T0)	n, median (25–75%)	18.0 (9.50–24.8) #2	14.5 (8.75–22.3)	20.5 (10.5–29.8) #1	0.105
VC% (T0)	n, median (25–75%)	59.0 (38.0–88.0) #1	38.0 (22.5–60.3)	81.0 (57.0–93.0)	** < 0.001**
PEF% (T0)	n, median (25–75%)	60.0 (47.0–86.0) #1	50.5 (35.3–65.5)	77.0 (51.0–89.0)	**0.006**
Ambulatory status	Ambulatory, n (%)	13 (26.0)	0	9 (39.1)	** < 0.001**
	Non-ambulatory, n (%)	37 (74.0)	22 (100)	14 (60.9)	

SMA type IV patients (n = 3) are not represented in a separate column. The number of missing data is indicated by the # symbol. Bold values in the table denote statistically significant differences. SMA, spinal muscular atrophy; *SMN2*, survival of motor neuron 2; RHS, Revised Hammersmith Functional Motor Scale; RULM, Revised Upper Limb Module; VC%, vital capacity percent predicted; PEF%, peak expiratory flow percent predicted

Patients' motor functions were assessed using RHS and RULM scores, which range from 0 to 69 and 0 to 37, respectively. Scores near 0 correspond to severe impairment, while higher scores indicate better motor function. The median (25th–75th percentile) RHS and RULM scores in the selected SMA cohort were 4.00 (1.50–16.0) and 18.0 (9.50–24.8), respectively. For respiratory function outcomes, the baseline VC% and PEF% values for SMA patients were 59.0 (38.0–88.0) and 60.0 (47.0–86.0), respectively. A total of 13 (26.0%) patients with SMA were ambulatory and 37 (74.0%) were non-ambulatory.

When comparing SMA type II and type III patients, significant differences were observed in *SMN2* copy number, RHS, VC%, PEF%, and ambulatory status (Table 1). Specifically, SMA type III patients had significantly higher *SMN2* copy numbers (*p* = 0.002), higher RHS scores (*p* = 0.003), higher VC% (*p* < 0.01) and PEF% (*p* = 0.006) values, and a higher likelihood of being ambulatory (*p* < 0.001). Age of the SMA type III group was significantly higher compared to the age of the SMA type II group (*p* < 0.001).

### Identification and Functional Analysis of miRNA–lncRNA–mRNA Regulatory Networks

**Fig. 1:**
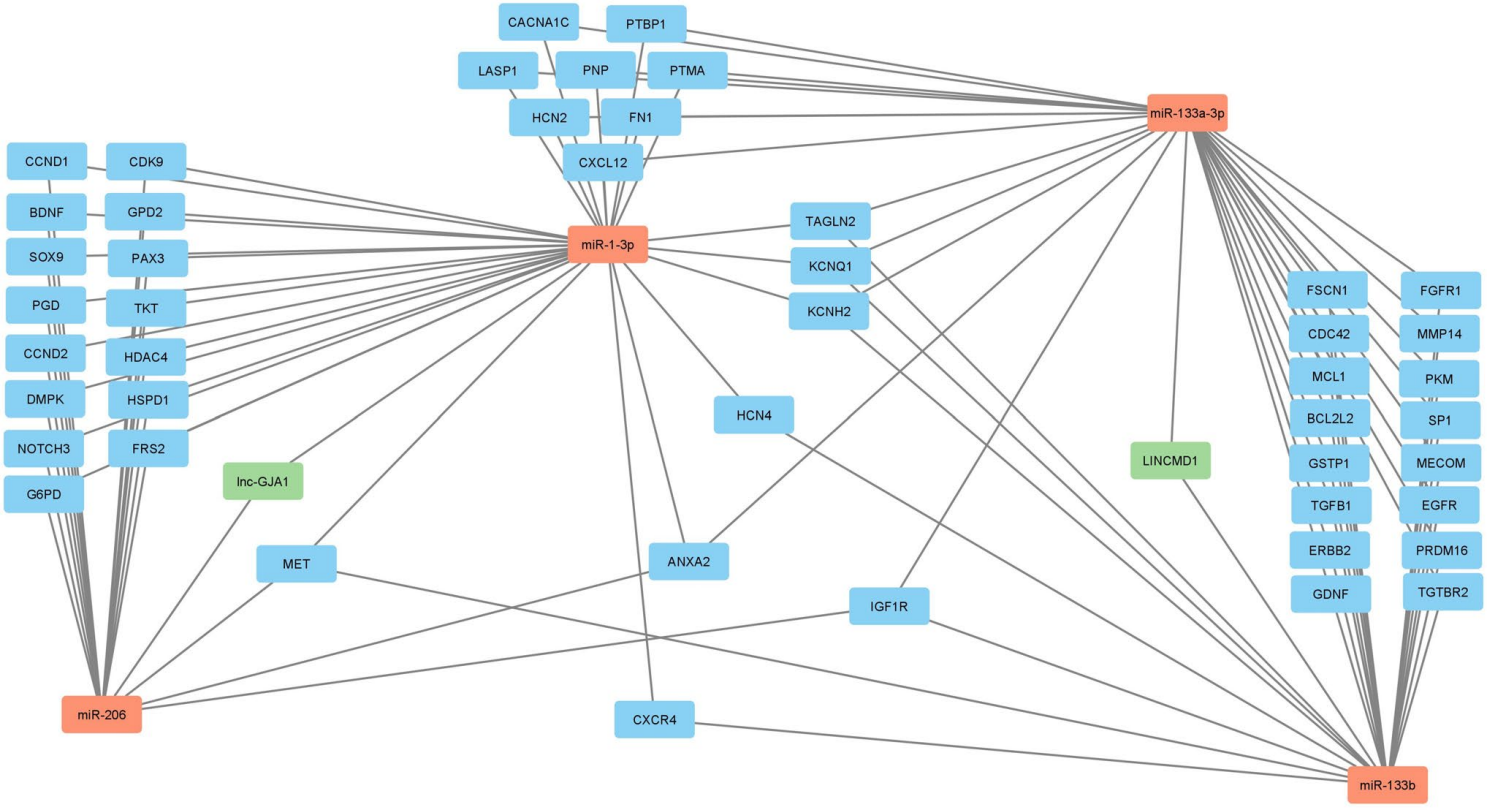
Visualization of the miRNA–lncRNA–mRNA interaction network. In the network, red nodes represent miRNAs, green nodes represent lncRNAs, blue nodes represent target mRNAs/genes, and the edges represent interactions between the nodes

Through a comprehensive literature search of the current state of knowledge, we identified four miRNAs (miR-1-3p, miR-133a-3p, miR-133b, and miR-206) as the most important candidates, as they are among the most frequently deregulated miRNAs between patients with SMA and unaffected controls, which can also be modulated by treatment [38]. Bioinformatics analysis of the regulatory interaction networks involving miRNAs revealed several clusters of predicted mRNA targets that are influenced by the interaction of two miRNAs, which in turn are linked by a common lncRNA (Fig. 1).

Functional enrichment analyses revealed specific signaling pathways associated with SMA. In particular, the hsa-miR-1-3p–*lnc-GJA1-2*–hsa-miR-206 axis was found to regulate the *PGD*, *G6PD*, and *TKT* genes involved in the pentose phosphate shunt associated with neuroinflammatory processes. Another notable axis, hsa-miR-133b–*LINCMD1*–hsa-miR-133a-3p, regulates the *FGFR1*, *SP1*, and *TGFB1* genes, which are associated with the positive regulation of vascular endothelial cell migration. These findings suggest that the identified axes may be involved in important regulatory pathways related to neuroinflammation and vascular regulation, the latter potentially contributing to SMA through microvascular pathology [54].

### Age- and Sex-dependent Expression of Selected miRNAs, Target mRNAs, and lncRNAs in Patients With SMA

We first analyzed the relative expression levels of selected RNAs at baseline and assessed the potential influence of demographic factors on RNA expression, including sex-specific differences and associations with the age of SMA patients. The age distribution between males and females did not differ significantly between the groups (*p* = 0.899).

**Fig. 2:**
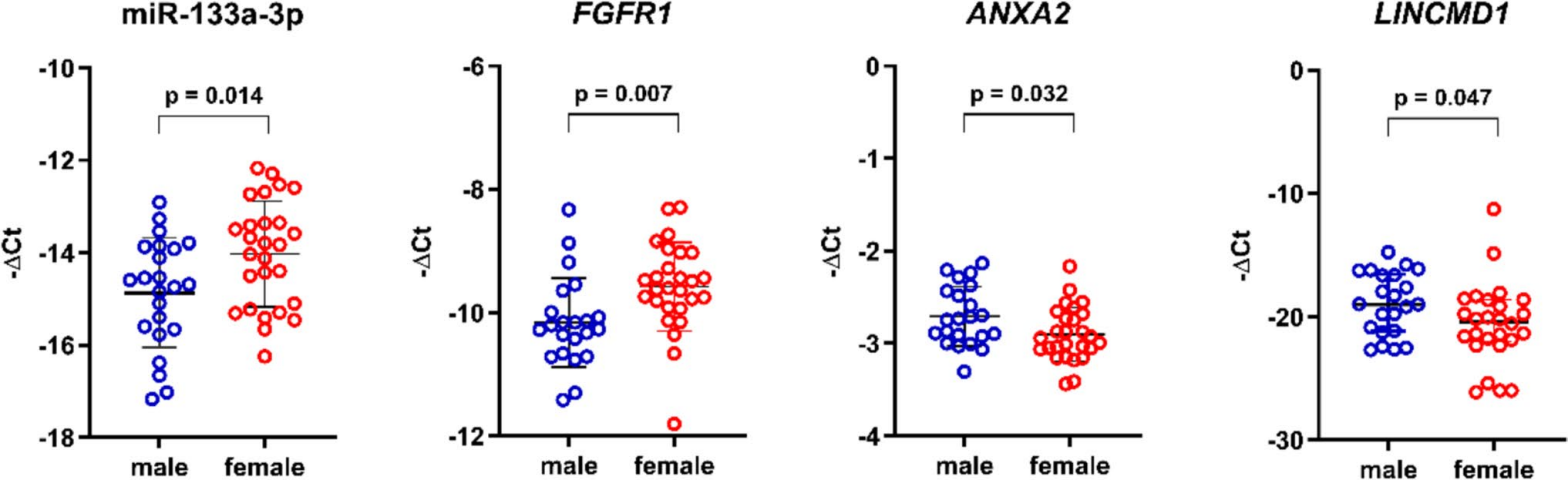
Sex-specific differences in relative RNA expression at baseline in patients with SMA (n = 50). Females showed significantly higher expression of miR-133a-3p and *FGFR1* compared to males (miR-133a-3p: *p* = 0.014; *FGFR1*: *p* = 0.007). Conversely, *ANXA2* and *LINCMD1* showed significantly higher expression in males compared to females (*ANXA2*: *p* = 0.032; *LINCMD1*: *p* = 0.047). miRNA levels were normalized to miR-16-5p and miR-191-5p, while the expression levels of target mRNAs and lncRNA were normalized using *ACTB* and *RPLP0* as reference genes. Gene expression is shown as log_2_-transformed 2^−ΔCt^ values (–ΔCt) for selected RNAs. A *p*-value < 0.05 was considered statistically significant. Normally distributed data are summarized as mean (SD) and compared using unpaired *t*-test; non-normally distributed data are presented as the median ± IQR and compared using the Mann–Whitney U test

With respect to sex-specific differences, significantly higher expression levels of miR-133a-3p (1.79-fold change, *p* = 0.014) and *FGFR1* (1.50-fold change, *p* = 0.007) were observed in females compared to males. In contrast, the expression levels of *LINCMD1* were significantly higher in males compared to females with a 2.64-fold (*p* = 0.047) difference. Similarly, expression levels of *ANXA2* were significantly higher in males by 1.15-fold (*p* = 0.032) (Fig. 2).

Correlation analysis revealed that the expression of *PGD* (*p* = 0.022, r = 0.326), *G6PD* (*p* = 0.017, r = 0.340), and *FGFR1* (*p* = 0.037, r = 0.299) positively correlated with the age of SMA patients. No significant correlations were observed between the expression of selected miRNAs or lncRNAs and the age of SMA patients (Table S2).

### Differential Expression of Selected miRNAs, Target mRNAs, and lncRNAs in Relation to SMA Type and Ambulatory Status

To assess the relationship between disease severity and RNA expression, we evaluated the expression levels of selected RNAs among SMA patients stratified by disease type (e.g., type II and type III) and ambulatory status (ambulatory vs. non-ambulatory). SMA type IV patients were excluded from the analysis due to the small sample size.

**Fig. 3:**
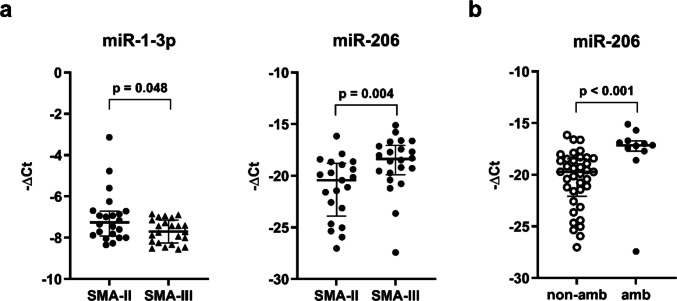
Differential expression of miRNAs in relation to SMA type (n = 45) and ambulatory status at baseline (n = 50). (**a**) miR-1-3p levels were significantly lower in SMA type III than in type II patients (*p* = 0.048), while miR-206 levels were significantly higher in SMA type III than in type II (*p* = 0.004). After the adjustment, the results were non-significant. (**b**) The expression of miR-206 was significantly higher in ambulatory patients compared to non-ambulatory patients (*p* < 0.001), and the difference remained significant after adjustment (*p* = 0.032). miRNA levels were normalized to miR-16-5p and miR-191-5p. Gene expression is shown as log_2_-transformed 2^−ΔCt^ values (–ΔCt). A *p*-value < 0.05 was considered statistically significant. Normally distributed data are summarized as mean (SD) and compared using unpaired *t*-test; non-normally distributed data are presented as the median ± IQR and compared using the Mann–Whitney U test. Amb, ambulatory patient; non-amb, non-ambulatory patient

Based on our results, miR-1-3p and miR-206 expression levels were significantly different between SMA type II and type III patients (Fig. 3a). The complete unadjusted results are shown in Supplementary Table S4. Whole blood miR-1-3p levels were significantly lower in type III compared to type II patients (1.24-fold, *p* = 0.048). On the contrary, miR-206 levels were significantly higher in type III than in type II patients (3.98-fold, *p* = 0.004). After adjusting for *SMN2* copy number, results were non-significant (miR-206: *p* = 0.185; miR-1-3p: *p* = 0.062), and the adjusted results are provided in Supplementary Table S5.

In addition, miR-206 expression was significantly different in SMA patients based on their ambulatory status (Fig. 3b), whereas the results for all analyzed RNAs are presented in Supplementary Table S6. 6.01-fold higher miR-206 expression levels were observed in ambulatory SMA patients (*p* < 0.001). After adjusting for age, disease duration, and *SMN2* copy number, the difference remained significant (*p* = 0.032), as shown in Supplementary Table S7. No significant differences were observed in the expression of other miRNAs or their predicted mRNA targets and lncRNA interactors between patients stratified by SMA type or ambulatory status (Tables S4–S7).

### Expression of Selected miRNAs, Target mRNAs, and lncRNAs in Relation to Motor Function in Patients With SMA

Further analysis of RNA expression was conducted in relation to the motor and upper limb functions of SMA patients at baseline, measured using standardized clinical scores (e.g., RHS, RULM). The analyses were adjusted for age, disease duration, and *SMN2* copy number (Table 2). In addition, the analyses were repeated after removing extreme RHS and RULM values to account for potential bias due to the floor and ceiling effects (Table 3). Tables S8–S10 summarize the data for all RNAs analyzed.

**Table 2:** Significant correlations of selected RNAs with RHS and RULM scores

RNA entity	RHS (n = 49)		RULM (n = 48)		RHS (n = 37)		RULM (n = 36)	
	**r**	***p*-value**	**r**	***p*-value**	**r**	***p***_**adj**_**-value**	**r**	***p***_**adj**_**-value**
miR-133a-3p	-0.443	0.001*	-0.450	**0.001**	-0.215	0.189	-0.380	0.089
miR-133b	-0.449	0.001*	-0.325	**0.024**	-0.192	0.241	-0.267	0.105
miR-206	0.656	< 0.001*	0.459	0.001*	0.430	**0.006**	0.389	**0.016**
*HDAC4* #1	-0.314	0.030*	-0.286	0.051	-0.233	-0.290	0.081	-0.290
*LINCMD1* #2	0.317	0.030*	0.164	0.320	0.201	0.233	0.285	0.092

The number of missing data is indicated by the # symbol. Bold values in the table denote statistically significant differences. Pearson correlation coefficient was used for normally distributed variables, and Spearman’s rank correlation coefficient* for non-normally distributed variables. For adjusted analyses, values are partial Pearson correlation coefficients, controlling for age, disease duration, and *SMN2* copy number. Adj, covariate-adjusted model; r, correlation coefficient; RHS, Revised Hammersmith Scale; RULM, Revised Upper Limb Module

**Table 3:** Significant correlations of selected RNAs with RHS and RULM scores after excluding ceiling and floor values

RNA entity	RHS (n = 38)		RULM (n = 41)		RHS (n = 29)		RULM (n = 30)	
	r	*p*-value	r	*p*-value	r	*p*_adj_-value	r	*p*_adj_-value
miR-133a-3p	-0.273	0.098*	-0.455	**0.003**	-0.136	0.466	-0.379	**0.032**
miR-133b	-0.204	0.219*	-0.404	**0.009**	-0.113	0.545	-0.455	**0.009**
miR-206	0.643	< 0.001*	0.315	0.045*	0.431	0.015	0.247	0.172

Bold values in the table denote statistically significant differences. Pearson correlation coefficient was used for normally distributed variables, and Spearman’s rank correlation coefficient* for non-normally distributed variables. For adjusted analyses, values are partial Pearson correlation coefficients, controlling for age, disease duration, and *SMN2* copy number. Adj, covariate-adjusted model; r, correlation coefficient; RHS, Revised Hammersmith Scale; RULM, Revised Upper Limb Module

RHS scores were significantly inversely correlated with the expression of miR-133a-3p (*p* = 0.001, r = -0.443), miR-133b (*p* = 0.001, r = -0.449), and *HDAC4* (*p* = 0.030, r = -0.314). In contrast, miR-206 (*p* < 0.001, r = 0.656) and *LINCMD1* (*p* = 0.030, r = 0.317) expression levels showed significant positive correlations with RHS scores. After adjustment, only the correlation between miR-206 expression and RHS remained significant (*p*_adj_ = 0.006, r = 0.430) (Table 2).

Similarly, significant inverse correlations were observed between RULM scores and miR-133a-3p expression (*p* = 0.001, r = -0.450) as well as miR-133b expression (*p* = 0.024, r = -0.325). Finally, correlation analysis revealed a positive correlation between miR-206 expression (*p* = 0.001, r = 0.459) and RULM scores, which remained significant after adjustment (*p*_adj_ = 0.016, r = 0.389) (Table 2).

To account for potential floor and ceiling effects, the analyses were conducted after excluding SMA patients with minimum or maximum RHS and RULM scores (Table 3). The expression of miR-206 exhibited a significant positive correlation with RHS scores (*p* < 0.001, r = 0.643), which remained significant after adjustment (*p* = 0.015, r = 0.431). For RULM scores, miR-133a-3p (*p* = 0.003, r = -0.455) and miR-133b (*p* = 0.009, r = -0.404) showed negative correlations, while miR-206 exhibited a significant positive correlation (*p* = 0.045, r = 0.315). After adjustment, the correlations between miR-133a-3p (*p* = 0.032, r = -0.379) and miR-133b (*p* = 0.009, r = -0.455) expression and RULM scores remained significant.

### Expression of Selected miRNAs, Target mRNAs, and lncRNAs in Relation to Pulmonary Function in Patients With SMA

To determine whether the expression levels of selected RNAs are associated with pulmonary function in SMA patients, we correlated their expression with VC% and PEF% at baseline (Table 4). The data for all RNAs analyzed are shown in Tables S8 and S9.

**Table 4:** Significant correlations of selected miRNAs with VC% and PEF%

RNA entity	VC% (n = 49)		PEF% (n = 49)		VC% (n = 37)		PEF% (n = 37)	
	**r**	***p*-value**	**r**	***p*-value**	**r**	***p***_**adj**_**-value**	**r**	***p***_**adj**_**-value**
miR-133a-3p	-0.471	** < 0.001**	-0.448	**0.001**	-0.430	**0.006**	-0.340	**0.034**
miR-133b	-0.393	**0.005**	-0.376	**0.008**	-0.379	**0.017**	-0.341	**0.033**
miR-206	0.543	< 0.001*	0.467	< 0.001*	0.428	**0.007**	0.346	**0.031**

Bold values in the table denote statistically significant differences. Pearson correlation coefficient was used for normally distributed variables, and Spearman’s rank correlation coefficient* for non-normally distributed variables. For adjusted analyses, values are partial Pearson correlation coefficients, controlling for age, disease duration, and *SMN2* copy number. Adj, covariate-adjusted model; r, correlation coefficient; VC%, vital capacity percent predicted; PEF%, peak expiratory flow percent predicted

The expression of miR-133a-3p was negatively correlated with both VC% (*p* < 0.001, r = -0.471) and PEF% (*p* = 0.001, r = -0.448). After adjusting for age, disease duration and *SMN2* copy number, the correlations remained significant (VC%: *p*_adj_ = 0.006, r = -0.430; PEF%: *p*_adj_ = 0.034, r = -0.340). Similarly, miR-133b expression showed a negative correlation with VC% and PEF% (VC%: *p* = 0.005, r = -0.393; PEF%: *p* = 0.008, r = -0.376) and the results remained significant after adjustment (VC%: *p*_adj_ = 0.017, r = -0.379; PEF%: *p*_adj_ = 0.033, r = -0.341). The expression levels of miR-206 were significantly positively correlated with VC% (*p* < 0.001, r = 0.543) and PEF% (*p* < 0.001, r = 0.467) and remained significant after adjustment (VC%: *p*_adj_ = 0.007, r = 0.428; PEF%: *p*_adj_ = 0.031, r = 0.346) (Table 4). No significant correlation was found between respiratory function and the expression of any of the investigated mRNA targets or lncRNAs (Tables S8, S9).

### Changes in the Expression Levels of Selected miRNAs, Target mRNAs, and lncRNAs During Treatment With Nusinersen and Risdiplam

**Fig. 4:**
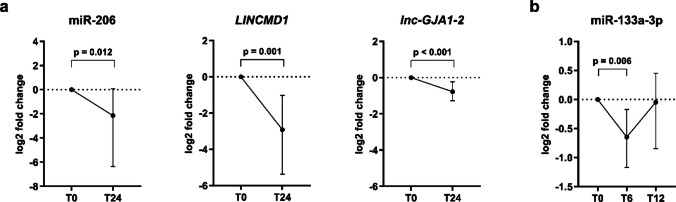
Expression of candidate RNAs in SMA patients at baseline (T0) and during SMN-restoring treatments. (**a**) During treatment with nusinersen, the expression levels of miR-206 (5.51-fold, *p* = 0.012), *LINCMD1* (6.48-fold, *p* = 0.001), and *lnc-GJA1-2* (1.70-fold, *p* < 0.001) decreased significantly between T0 and T24. (**b**) During risdiplam treatment, miR-133a-3p (1.60-fold, *p* = 0.006) levels decreased significantly at T6 compared to baseline. Relative quantification of lncRNAs was based on the 2^−ΔΔCt^ method with *ACTB* and *RPLP0* as reference genes for normalization, while miRNA levels were normalized to miR-16-5p and miR-191-5p. Expression values are presented as log_2_-fold change, where time point T0 (before treatment) was used as a calibrator for ΔΔCt and is represented by a dotted line in the graphs. Only results with a fold change of ≥ 1.5 and a *p*-value of < 0.05 are presented. Normally distributed data are summarized as the mean (SD) and compared with a one-sample *t*-test. Non-normally distributed data are summarized as the median ± IQR and compared using a Wilcoxon signed-rank test

We also analyzed within-patient changes in expression levels from baseline to follow-up during SMN-restoring treatment (Fig. 4). Analyses were conducted separately for the nusinersen and risdiplam groups, using the same RNA panel in both groups.

Comparative analysis demonstrated treatment-specific expression patterns. Expression levels of miR-206, *LINCMD1*, *lnc-GJA1-2*, and several target mRNAs in nusinersen-treated patients were consistently downregulated after nusinersen treatment. Specifically, the expression levels of miR-206 (5.51-fold decrease, *p* = 0.012), *LINCMD1* (6.48-fold decrease, *p* = 0.001) and *lnc-GJA1-2* (1.70-fold decrease, *p* < 0.001) decreased significantly between baseline (T0) and T24 (Fig. 4a). Several mRNA targets, including *PGD* (1.22-fold decrease, *p* = 0.004), *G6PD* (1.20-fold decrease, *p* = 0.001), *TKT* (1.24-fold decrease, *p* = 0.001), *HDAC4* (1.11-fold decrease, *p* = 0.027), *SP1* (1.21-fold decrease, *p* = 0.003), *TGFB1* (1.10-fold decrease, *p* = 0.003), *KCNQ1* (1.18-fold decrease, *p* < 0.001), and *IGF1R* (1.30-fold decrease, *p* < 0.001) also showed modest but significant downregulation between T0 and T24 (Fig. S1).

In contrast, risdiplam treatment resulted in a significant downregulation of only miR-133a-3p at T6 (1.60-fold decrease, *p* = 0.006). However, no significant changes were detected regarding the expression of other miRNAs, target mRNAs, or lncRNAs (Fig. 4b). At time point T12, no significant differences were observed in the expression levels of any of the RNAs analyzed.

### Expression of SMN Transcript Variants in Relation to Demographic and Clinical Characteristics of Patients With SMA

To further clarify the molecular effects of SMN-restoring treatments, we examined the expression levels of key SMN transcripts in relation to sex, age, *SMN2* copy number, SMA type, ambulatory status, motor function, and pulmonary function in patients with SMA.

No significant sex-specific differences or age-related correlations were observed with respect to the expression levels of *SMN*-total, *SMN*-FL, or *SMN*-∆7, nor in the *SMN*-FL/*SMN*-∆7 ratio (Tables S2, S3).

Regarding associations with disease severity, expression levels of *SMN*-total, *SMN*-FL, and *SMN*-∆7 showed a trend toward higher expression in SMA type III compared to type II patients. However, only *SMN*-∆7 expression levels were statistically significantly higher in SMA type III compared to type II patients (1.22-fold, *p* = 0.040), although this difference was not significant after adjustment for *SMN2* copy number (Table S4, S5).

To further investigate the impact of *SMN2* copy number on *SMN* transcript expression, we compared the expression levels of *SMN* transcripts in SMA patients with three and four copies of *SMN2* (Table S11). Patients with two or five *SMN2* copies were excluded from the analyses due to the insufficient group sizes. Patients with four *SMN2* copies showed significantly higher expression of *SMN*-total (1.26-fold, *p* = 0.017), *SMN*-FL (1.36-fold, *p* = 0.005), and *SMN*-∆7 (1.30-fold, *p* = 0.005) compared to patients with three *SMN2* copies.

In addition, *SMN*-total, *SMN*-FL, and *SMN*-∆7 showed significant differences in SMA patients stratified into ambulatory and non-ambulatory groups. Higher expressions of *SMN*-total (1.40-fold, *p* = 0.006), *SMN*-FL (1.44-fold, *p* = 0.006), and *SMN*-∆7 (1.47-fold, *p* = 0.003) were observed in ambulatory compared to non-ambulatory patients. After adjusting for age, disease duration, and *SMN2* copy number, the differences remained significant (*SMN*-total: *p* = 0.016; *SMN*-FL: *p* = 0.008; *SMN*-∆7: *p* = 0.023; Tables S6, S7).

While the *SMN*-FL/*SMN*-∆7 ratio differed significantly between type II and type III after adjusting for *SMN2* copy number (1.10-fold, *p* = 0.038), it was non-significant in the unadjusted analyses (*p* = 0.082; Table S2). Furthermore, no statistically significant differences in the *SMN*-FL/*SMN*-∆7 ratio were observed between patients stratified by *SMN2* copy number or ambulatory status (Tables S4 –S7), suggesting that splicing efficiency, as reflected by the *SMN*-FL/*SMN*-∆7 ratio, does not systematically differ between groups.

We further analyzed the expression of *SMN* transcripts in relation to motor function, upper limb function, and pulmonary function in patients with SMA at baseline, using RHS, RULM, VC%, and PEF% (Tables 5, 6, and 7). The results showed that RHS scores were significantly positively correlated with the expression of *SMN*-total (*p* = 0.004, r = 0.408), *SMN*-FL (*p* < 0.001, r = 0.494), and *SMN*-∆7 (*p* < 0.001, r = 0.637; Table 5). The correlation remained significant after adjustment for age, disease duration, and *SMN2* copy number (*SMN*-total: *p*_adj_ = 0.033; *SMN*-FL: *p*_adj_ = 0.005; *SMN*-∆7: *p*_adj_ = 0.013). Similarly, expression levels of *SMN*-total (*p* = 0.045, r = 0.294), *SMN*-FL (*p* = 0.009, r = 0.379), and *SMN*-∆7 (*p* < 0.001, r = 0.502) showed significant positive correlations with RULM scores. After adjustment, only the correlation between *SMN*-∆7 expression and RULM remained significant (*p*_adj_ = 0.040).

**Table 5:** Significant correlations of *SMN* transcripts with RHS and RULM scores

RNA entity	RHS (n = 48)		RULM (n = 47)		RHS (n = 36)		RULM (n = 35)	
	r	*p*-value	r	*p*-value	r	*p*_adj_-value	r	*p*_adj_-value
*SMN*-total	0.408	0.004*	0.294	0.045*	0.347	**0.033**	0.265	0.113
*SMN*-FL	0.494	< 0.001*	0.379	**0.009**	0.442	**0.005**	0.247	0.140
*SMN*-∆7	0.637	< 0.001*	0.502	** < 0.001**	0.398	**0.013**	0.339	**0.040**

Bold values in the table denote statistically significant differences. Pearson correlation coefficient was used for normally distributed variables, and Spearman’s rank correlation coefficient* for non-normally distributed variables. For adjusted analyses, values are partial Pearson correlation coefficients, controlling for age, disease duration, and *SMN2* copy number. Adj, covariate-adjusted model; r, correlation coefficient; RHS, Revised Hammersmith Scale; RULM, Revised Upper Limb Module

**Table 6:** Significant correlations of *SMN* transcripts with RHS and RULM scores after excluding ceiling and floor values

RNA entity	RHS (n = 37)		RULM (n = 40)		RHS (n = 28)		RULM (n = 29)	
	r	*p*-value	r	*p*-value	r	*p*_adj_-value	r	*p*_adj_-value
*SMN*-total	0.576	< 0.001*	0.212	0.189*	0.376	**0.040**	0.114	0.543
*SMN*-FL	0.538	< 0.001*	0.253	0.115	0.413	**0.023**	0.022	0.908
*SMN*-∆7	0.596	< 0.001*	0.298	0.062	0.397	**0.030**	0.156	0.403

Bold values in the table denote statistically significant differences. Pearson correlation coefficient was used for normally distributed variables, and Spearman’s rank correlation coefficient* for non-normally distributed variables. For adjusted analyses, values are partial Pearson correlation coefficients, controlling for age, disease duration, and *SMN2* copy number. Adj, covariate-adjusted model; r, correlation coefficient; RHS, Revised Hammersmith Scale; RULM, Revised Upper Limb Module

**Table 7:** Significant correlations of SMN transcripts with VC% and PEF%

RNA entity	VC% (n = 48)		PEF% (n = 48)		VC% (n = 40)		PEF% (n = 40)	
	r	*p*-value	r	*p*-value	r	*p*_adj_-value	r	*p*_adj_-value
*SMN*-total	0.318	0.028*	0.295	0.042*	0.251	0.128	0.297	0.070
*SMN*-FL	0.370	**0.010**	0.423	**0.003**	0.297	0.070	0.396	**0.014**
*SMN*-∆7	0.506	** < 0.001**	0.515	** < 0.001**	0.293	0.074	0.323	**0.048**

Bold values in the table denote statistically significant differences. Pearson correlation coefficient was used for normally distributed variables, and Spearman’s rank correlation coefficient* for non-normally distributed variables. For adjusted analyses, values are partial Pearson correlation coefficients, controlling for age, disease duration, and *SMN2* copy number. Adj, covariate-adjusted model; r, correlation coefficient; VC%, vital capacity percent predicted; PEF%, peak expiratory flow percent predicted

After excluding extreme RHS and RULM values from the analyses, significant correlations were observed between the RHS scores and the expression levels of *SMN*-total (*p* < 0.001, r = 0.576), *SMN*-FL (*p* < 0.001, r = 0.538), and *SMN*-∆7 (*p* < 0.001, r = 0.596; Table 6). After adjustment, the correlations remained significant (*SMN*-total: *p*_adj_ = 0.040; *SMN*-FL: *p*_adj_ = 0.023; *SMN*-∆7: *p*_adj_ = 0.030). Interestingly, after exclusion of extreme values, RULM scores showed no significant correlations with the expression levels of *SMN*-total, *SMN*-FL, or *SMN*-∆7.

Finally, correlation analysis revealed a positive correlation between VC% and *SMN*-total (*p* = 0.028, r = 0.318), *SMN*-FL (*p* = 0.010, r = 0.370), and *SMN*-∆7 (*p* < 0.001, r = 0.506; Table 7). Similarly, *SMN*-total (*p* = 0.042, r = 0.295), *SMN*-FL (*p* = 0.003, r = 0.423), and *SMN*-∆7 (*p* < 0.001, r = 0.515) expression levels showed significant correlations with PEF%. After adjustment, only the correlations between *SMN*-FL (*p*_adj_ = 0.014) and *SMN*-∆7 (*p*_adj_ = 0.048) expression levels and PEF% remained significant.

### Changes in the Expression Levels of *SMN* Transcript Variants During Treatment With Nusinersen and Risdiplam

We further analyzed *SMN* transcript expression changes during SMN-restoring treatment, with separate analyses for nusinersen and risdiplam (Tables S12–S14).

In the nusinersen-treated group, longitudinal analysis revealed distinct expression patterns, including a modest but statistically significant downregulation of *SMN*-total (1.15-fold decrease, *p* = 0.016) and *SMN*-∆7 (1.10-fold decrease, *p* = 0.039) from baseline to T24.

**Fig. 5:**
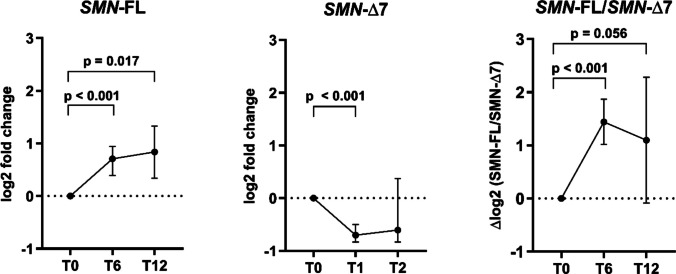
Expression of *SMN* transcript variants in SMA patients at baseline (T0) and during SMN-restoring treatment. During risdiplam treatment, the expression levels of *SMN*-FL increased significantly between T0 and T6 (1.65-fold; *p* < 0.001) and between T0 and T12 (1.68-fold, *p* = 0.017). Levels of *SMN*-∆7 decreased significantly at T6 compared to baseline (1.66-fold, *p* < 0.001). At T6, the *SMN*-FL/*SMN*-∆7 ratio increased significantly (2.72-fold, *p* < 0.001) relative to T0. Relative quantification of *SMN* transcript variants was based on the 2^−ΔΔCt^ method with *ACTB* and *RPLP0* as reference genes for normalization. Expression values are presented as log2-fold change, where time point T0 (before treatment) was used as a calibrator for ΔΔCt and is represented by a dotted line in the graphs. Only results with a fold change of ≥ 1.5 and a *p*-value of < 0.05 are presented. Normally distributed data are summarized as the mean (SD) and compared with the one-sample *t*-test. Non-normally distributed data are summarized as the median ± IQR and compared using the Wilcoxon signed-rank test

In contrast, our data show that risdiplam administration resulted in a significant upregulation of *SMN*-FL at T6 (1.65-fold increase, *p* < 0.001) as well as at T12 (1.68-fold increase, *p* = 0.017). On the contrary, *SMN*-∆7 expression was significantly decreased at T6 (1.66-fold decrease, *p* < 0.001). In addition, the ratio of *SMN*-FL/*SMN*-∆7 showed a significant increase between T0 to T6, corresponding to a 2.72-fold increase (*p* < 0.001). At T12, the *SMN*-FL/*SMN*-∆7 ratio showed a trend toward statistical significance (*p* = 0.086; Fig. 5).

### Correlation Analysis of miRNAs and Corresponding mRNA Targets, lncRNAs, and *SMN* Transcript Variants in SMA Patients

To further validate the regulatory relationships within the predicted RNA networks, we assessed expression-level correlations among selected RNAs (Table 8). We also examined pairwise correlations among studied miRNAs, as they are assumed to belong to the same miRNA cluster [39], as well as between miRNAs and *SMN* transcripts.

**Table 8:** Significant correlations between miRNAs and corresponding target mRNAs, lncRNAs, and *SMN* transcripts (n = 50)

RNA interactor	miR-1-3p		miR-133a-3p		miR-133b		miR-206	
	r	*p*-value	r	*p*-value	r	*p*-value	r	*p*-value
miR-133a-3p	0.309	0.029*	/	/	/	/	/	/
miR-133b	0.425	0.002*	0.756	** < 0.001**	/	/	/	/
*HDAC4* #1	0.256	0.076*	/	/	/	/	-0.350	0.014*
*FGFR1* #1	/	/	0.342	**0.016**	0.405	**0.004**	/	/
*lnc-GJA1-2* #2	0.324	0.025*	/	/	/	/	-0.092	0.543*
*SMN*-total	0.143	0.326*	**0.049**	0.736*	0.175	0.229*	0.361	0.011*
*SMN*-FL	**0.013**	0.928*	-0.078	0.596	0.127	0.384	0.368	0.009*
*SMN*-∆7	-0.128	0.381*	-0.177	0.224	-0.009	0.953	0.494	< 0.001*

The number of missing data is indicated by the # symbols. Bold values in the table denote statistically significant differences. Pearson correlation coefficient was used for normally distributed variables, and Spearman’s rank correlation coefficient* for non-normally distributed variables

Significant positive correlations were found between miR-1-3p and miR-133a-3p (*p* = 0.029, r = 0.309), and with miR-133b (*p* = 0.002, r = 0.425). Similarly, a positive correlation was found between miR-1-3p and *lnc-GJA1-2* (*p* = 0.025, r = 0.324).

We observed a significant positive correlation between miR-133a-3p and miR-133b (*p* < 0.001, r = 0.756). Furthermore, both miR-133a-3p and miR-133b showed significant positive correlations with their putative target *FGFR1* (miR-133a-3p: *p* = 0.016, r = 0.342; miR-133b: *p* = 0.004, r = 0.405).

Lastly, correlation analysis revealed a significant negative correlation between miR-206 and its predicted target *HDAC4* (*p* = 0.014, r = -0.350). Regarding correlations with *SMN* transcript variants, positive correlations were observed between miR-206 and all three *SMN* variants (*SMN*-total: *p* = 0.011, r = 0.361; *SMN*-FL: *p* = 0.009, r = 0.368; *SMN*-∆7: *p* < 0.001, r = 0.494). The complete results of expression-level correlation analysis are presented in Supplementary Table S15.

## Discussion

In this study, we investigated selected circulating RNAs and *SMN* transcript variants in whole blood as potential minimally invasive biomarkers of clinical status and treatment-associated molecular changes in adult SMA patients, and whether their patterns offer insight into disease mechanisms. We examined their associations with disease phenotype, motor and respiratory function, and longitudinal changes during nusinersen and risdiplam treatment.

Among the RNAs studied, miR-206 emerged as the most clinically informative blood-based marker of disease severity and functional status in SMA. At baseline, higher miR-206 expression was observed in patients with milder phenotypes and in ambulatory patients. It also showed the most consistent associations with motor and respiratory function, remaining significant after adjustment for covariates and after accounting for floor and ceiling effects. Together, these results suggest that circulating miR-206 levels may reflect preserved functional capacity and residual muscle integrity in SMA and may also be influenced by compensatory regenerative activity. Higher expression in milder and ambulatory patients may therefore be consistent with better preserved muscle reserve and a more effective response to denervation. These observations are also consistent with a potential neuroprotective role of miR-206 in the survival and function of MNs and muscle cells. Indeed, miR-206 is expressed not only in skeletal muscle [55], but also in spinal MNs [56]. Its protective role is further supported by experimental studies in which miR-206 has been reported to regulate neurodegenerative pathways and to be associated with attenuated MN degeneration and a milder disease phenotype [57, 58]. Another recent study in SMN-Δ7 mice found that loss of SMN leads to downregulation of miR-206, while its supplementation restored muscle function and myotube formation [59]. Consistent with experimental observations, our results support a possible relationship between SMN-related molecular status and miR-206 expression, as miR-206 levels were lower in SMA type II patients carrying significantly fewer *SMN2* copies than in type III patients and showed positive correlations with *SMN*-total, *SMN*-FL, and *SMN*-∆7. Similar protective effects of miR-206 have been described in amyotrophic lateral sclerosis, where miR-206 was shown to promote the regeneration of neuromuscular synapses and to delay disease onset and progression [60–64]. Published human data on miR-206 in SMA, however, remain heterogeneous. Catapano et al. found no significant correlation of miR-206 serum levels with motor performance and reported no significant difference in miR-206 expression between SMA type II and type III patients aged 4–14 years or when comparing SMA patients with healthy controls [41]. Another study, however, reported increased serum miR-206 levels in SMA type II and III patients (aged 6.86 ± 3.33 years), suggesting a potential diagnostic value of miR-206 levels [40].

During nusinersen treatment, miR-206 expression decreased significantly over 24 months, consistent with the direction of change reported by Bonanno et al., who observed a tendency towards reduction in miR-206 levels after 6 months in serum samples from infantile patients with SMA type II and type III [42]. Another study found that higher CSF miR-206 levels predicted a poorer response to nusinersen, as they inversely correlated with changes in motor function scores; however, the authors observed no correlation between CSF miR-206 levels and baseline motor scores [43]. Due to the predominantly CNS-limited effects of nusinersen, the changes observed in blood are more likely to reflect indirect downstream effects of treatment than direct peripheral effects. Therefore, decreased circulating miR-206 levels during treatment may be related to reduced ongoing muscle stress and reduced need for regenerative activity as treatment stabilizes the neuromuscular system. Indeed, evidence indicates that nusinersen may contribute to the restoration of muscle homeostasis indirectly by supporting the essential role of SMN in maintaining MNs and neuromuscular junctions, thereby limiting further muscle denervation and potentially promoting reinnervation [65, 66].

Alongside miR-206, *SMN* transcript measures emerged as potentially informative molecular biomarkers in our study, particularly in relation to *SMN2* copy number, functional status, and treatment-associated molecular changes. Total *SMN* transcripts and both transcript variants showed a trend towards higher expression in SMA patients with type III compared to type II, although only *SMN*-∆7 reached statistical significance. However, this difference became non-significant after adjustment for *SMN2* copy number, suggesting that the observed difference was primarily driven by *SMN2* copy number. Consistently, higher expression of all three *SMN* transcript measures was observed both in patients with more *SMN2* copies and in ambulatory compared with non-ambulatory patients, even after adjustment for age, disease duration, and *SMN2* copy number. Together, these findings suggest that *SMN* transcript abundance reflects functional status in SMA, which is strongly influenced by genetically determined *SMN2* dosage. As noted by Giorgia et al. [67], previous studies have reported conflicting findings regarding the value of *SMN* transcripts as biomarkers of disease severity. Tiziano et al. [68] reported higher *SMN2*-FL levels in milder phenotypes. Similarly, Crawford et al. [69] described higher *SMN2*-FL and *SMN*-∆7 levels in milder phenotypes, while the *SMN2*-FL/*SMN*-∆7 ratio remained unchanged. Vezain et al. [70] also observed a weak inverse correlation between *SMN*-FL and *SMN*-∆7 transcript levels and disease severity; however, when *SMN2* copy number was taken into account, transcript levels did not differ. In the same study, both *SMN*-FL and *SMN*-∆7 increased with *SMN2* copy number, whereas the ratio between the two transcripts remained the same across SMA types and *SMN2* copy numbers, consistent with our findings. Similarly, Sumner et al. [71] reported increased *SMN*-FL levels with higher *SMN2* copy number, while several other studies found no significant differences in the expression of *SMN*-FL or *SMN*-∆7 between SMA types, or no correlation with *SMN2* copy number [69, 72, 73].

To further assess the potential of *SMN* transcript variants as biomarkers of functional status, we examined their associations with motor and pulmonary function. Higher expression of *SMN*-total, *SMN*-FL, and *SMN*-∆7 was associated with better motor function, with the most robust adjusted association observed for *SMN*-∆7. Positive associations were also observed with pulmonary function. Our findings are consistent with those of Tiziano et al. [72], who reported a correlation between motor performance and *SMN2*-FL expression, particularly in ambulatory SMA patients. Crawford et al. [69] likewise observed a modest association between *SMN2*-FL and motor function scores, although this trend was not seen after excluding patients with floor and ceiling scores, which supports our approach of conducting comparable sensitivity analyses on the RHS and RULM scales.

Longitudinally, nusinersen was associated with a modest decrease in *SMN*-∆7 and, consequently, in *SMN*-total, whereas *SMN*-FL remained unchanged, suggesting that peripheral blood *SMN* transcript levels may not fully reflect CNS-specific treatment-induced dynamics [74]. In contrast, Trifunov et al. [75] reported increased *SMN*-FL transcripts in serum extracellular vesicles (EVs) after 14 months of nusinersen treatment, possibly reflecting higher sensitivity of EV-enriched RNA for detecting treatment-related molecular changes. For risdiplam, however, we observed the expected splicing shift, with significantly increased *SMN*-FL and *SMN*-FL/*SMN*-∆7 and simultaneously decreased *SMN*-∆7 after 6 months of treatment. After 12 months, *SMN*-FL remained significantly increased, whereas the *SMN*-FL/*SMN*-∆7 ratio showed only a trend toward significance. Unlike intrathecal nusinersen, orally administered risdiplam is distributed to both the CNS and peripheral tissues, including blood, where it increases functional SMN protein levels [76]. These findings are therefore consistent with its known mechanism of promoting *SMN2* splicing toward full-length transcripts [77].

Our data also suggest a potentially relevant miR-133a-3p–*LINCMD1*–miR-133b regulatory axis in SMA. Both miRNAs were strongly correlated with each other and showed inverse associations with motor function, particularly upper limb function, and pulmonary function at baseline. Longitudinally, nusinersen treatment was associated with significantly decreased *LINCMD1* expression after 24 months, while miR-133a-3p decreased after 6 months of risdiplam treatment. Our findings are directionally consistent with a previous study by Bonanno et al., who reported significantly reduced miR-133a and miR-133b after 6 months of nusinersen treatment, with miR-133a also significantly associated with clinical improvement in SMA type II and III patients [42]. Together with the inverse associations of miR-133a-3p with motor function, these findings align with previous data demonstrating miR-133 decline during muscle regeneration processes [78–81], suggesting the potential of miR-133 as a marker of skeletal muscle recovery.

Mechanistically, *LINCMD1* supports the biological plausibility of this axis, as it is a muscle-specific lncRNA involved in muscle differentiation and acts as a competing endogenous RNA for miR-133 targets [82]. Additionally, *LINCMD1* has been identified as a host transcript for miR-133b, rendering their biogenesis mutually exclusive [83]. Previous studies have also proposed that increased *LINCMD1* expression may represent a compensatory response to skeletal muscle atrophy [84, 85]; therefore, its decrease during nusinersen treatment may reflect a reduced need for muscle regeneration as effective therapy stabilizes MNs and limits further muscle atrophy. Finally, *FGFR1* emerged as a plausible downstream effector of the miR-133a-3p–*LINCMD1*–miR-133b axis, as its expression was associated with both miRNAs in our dataset, and enrichment analysis linked the axis to genes involved in the positive regulation of vascular endothelial cell migration, including *FGFR1*. This interpretation is further supported by evidence that miR-133 can directly downregulate *FGFR1* and thus modulate endothelial cell proliferation and migration [86]. Together, these findings suggest that this network may contribute to SMA through both myogenic and microvascular mechanisms, which is consistent with recent human evidence of microvasculopathy in SMA [54, 87] and with reports of early dysregulation of FGF/FGFR1 signaling in SMA models [88]. Although this regulatory axis was not directly validated in our study, the observed expression patterns and enrichment results support its biological plausibility in SMA.

In our study, the miR-1-3p–*lnc-GJA1-2*–miR-206 axis emerged as a potentially relevant regulatory axis linked to the *PGD*, *G6PD* and *TKT* genes, which were associated with the pentosephosphate pathway (PPP) in the enrichment analysis. Longitudinal analysis showed significantly decreased *lnc-GJA1-2* expression at 24 months after the start of nusinersen treatment, as well as a modest but significant reduction in the expression levels of these genes. These findings are potentially biologically relevant because *PGD*, *G6PD,* and *TKT* are involved in the PPP, the primary source of NADPH, which is essential for neuronal survival [89, 90] and protection against oxidative stress, a process implicated in the pathology of neurodegenerative diseases, including SMA [91]. Indeed, increased G6PD activity and RNA content have previously been reported in various neuromuscular diseases, including SMA [92], and PPP-related enzymes, including G6PD and PGD, have been linked to the maintenance of reduced glutathione in damaged muscle fibers [93], supporting the role of the PPP in antioxidant defense. Accordingly, the decline in this axis during nusinersen treatment may be compatible with reduced demand for antioxidant defense and tissue repair. Although the potential role of *lnc-GJA1-2* in muscle physiology or SMA pathogenesis remains to be established, previous evidence that miR-1 and miR-206 regulate GJA1/connexin43 during skeletal muscle development supports the plausibility of a GJA1-associated regulatory context for this axis [94].

Notwithstanding the insightful information provided by this study, certain limitations must be acknowledged. First, although whole blood is a practical and minimally invasive source for biomarker discovery, the measured RNA profiles, including *SMN* transcript variants, may not fully reflect molecular processes in disease-relevant tissues, including MNs and skeletal muscles, which limits mechanistic interpretation. Second, the cohort size was modest, particularly for stratified and longitudinal analyses, which may have limited the ability to detect subtle differences in clinical characteristics and RNA expression levels. Third, the minimal longitudinal change in functional scores prevented objective differentiation between responders and non-responders to therapy, thereby limiting assessment of the predictive or response-biomarker potential of the analyzed RNAs. Importantly, however, minimal changes in functional scores do not necessarily indicate a lack of treatment effect, as stabilization itself may reflect clinical benefit in SMA, where untreated patients typically show progressive functional decline [95]. Finally, future studies should also include pediatric and SMA type I patients, as nusinersen has been shown to improve clinical outcomes more clearly in children compared to the variable outcomes in late-onset SMA [14, 96]. In addition, inclusion of patients treated with onasemnogene abeparvovec gene therapy would allow evaluation of selected RNA-based biomarkers across different SMA treatments. Further work should include functional validation and more detailed characterization of the discussed RNA networks, including transcriptome-wide approaches that may help identify additional molecular biomarkers relevant for monitoring disease progression and treatment response.

In summary, our findings provide new insights into the molecular landscape of SMA, contribute to a better understanding of the complex pathophysiology of the disease, and offer a basis for future SMA biomarker research.

## Supplementary Information

Below is the link to the electronic supplementary material.

Supplementary file1 (DOCX 220 KB)

## Data Availability

A de-identified patient-level dataset relevant to the findings of this study is available from the corresponding author upon reasonable request. Summary data supporting the findings are included in the article and Supplementary Material.
