# Supplementary material for: MyomiR Networks in Spinal Muscular Atrophy: Associations With Clinical Severity and Treatment Response
Source: Mol Neurobiol. 2026 Apr 30;63(1):601. doi: 10.1007/s12035-026-05862-4 (PMC13132963; doi:10.1007/s12035-026-05862-4)
Supplement: Supplementary file 1 — Supplementary file1 (DOCX 220 KB) [file 12035_2026_5862_MOESM1_ESM.docx]

**Supplementary**

**
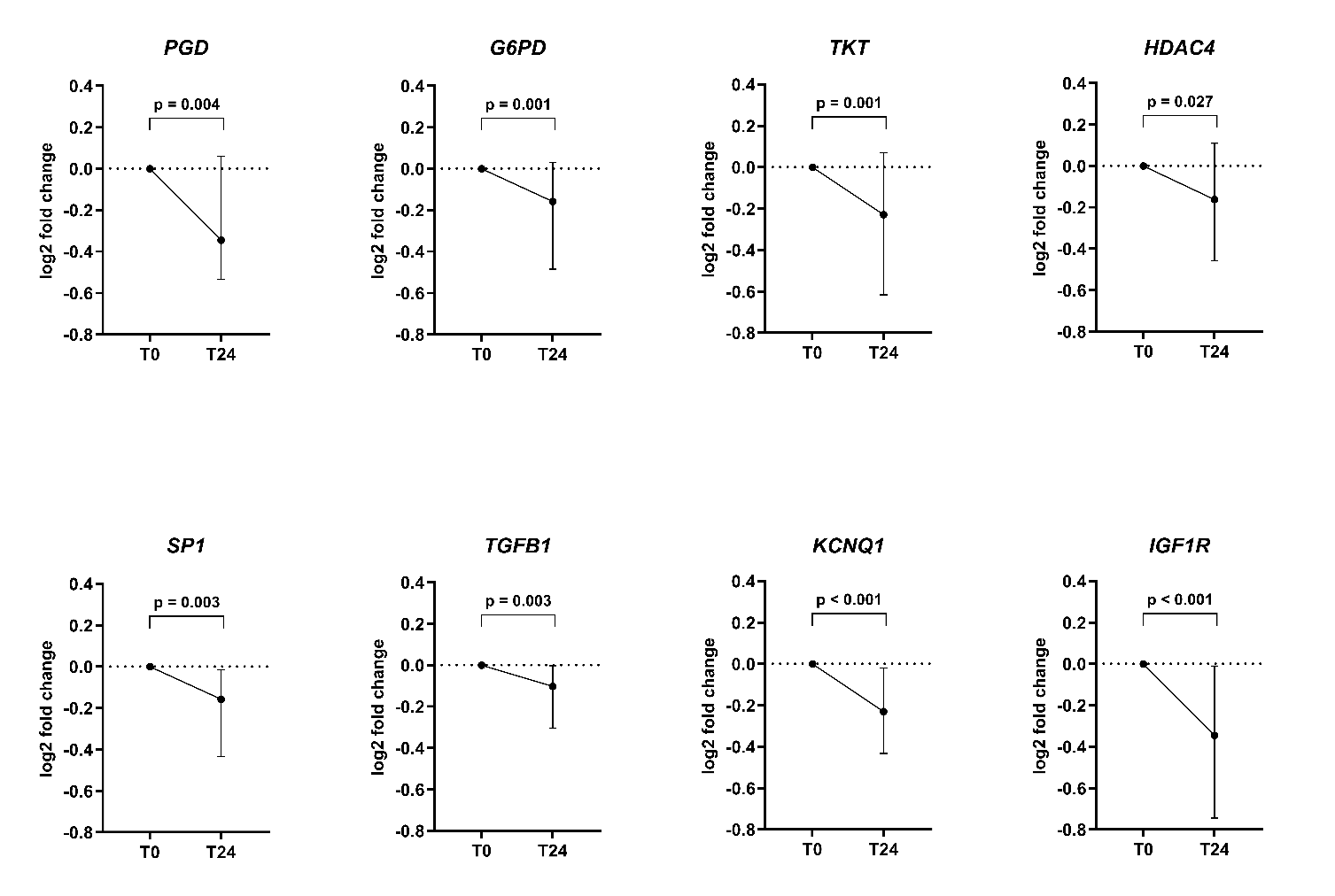
**

**Fig. S1** **Expression of target mRNAs in SMA patients at baseline (T0) and during SMN-restoring treatments.** During treatment with nusinersen, the expression levels of *PGD* (1.22-fold decrease, *p* = 0.004), *G6PD* (1.20-fold decrease, *p* = 0.001), *TKT* (1.24-fold decrease, *p* = 0.001), *HDAC4* (1.11-fold decrease, *p* = 0.027), *SP1* (1.21-fold decrease, *p* = 0.003), *TGFB1* (1.10-fold decrease, *p* = 0.003), *KCNQ1* (1.18-fold decrease, *p* < 0.001) and *IGF1R* (1.30-fold decrease, *p* < 0.001) decreased significantly between T0 and T24. Relative quantification of mRNAs was based on the 2^−ΔΔCt^ method with *ATCB* and *RPLP0* as reference genes for normalization. Expression values are presented as log_2_-fold change, where time point T0 (before treatment) was used as a calibrator for ΔΔCt and is represented by a dotted line in the graphs. Only results with a *p*-value of < 0.05 are presented. Normally distributed data are summarized as the mean (SD) and compared with a one-sample t-test. Non-normally distributed data are summarized as the median ± IQR and compared using a Wilcoxon signed-rank test

**Table S1**. TaqMan® Gene Expression Assays used for RT-qPCR.

| **Gene/miRNA symbol** | **Gene/miRNA name** | **Assay ID** |
| --- | --- | --- |
| miR-1-3p | hsa-miRNA-1-3p | 477820_mir |
| miR-133a-3p | hsa-miRNA-133a-3p | 478511_mir |
| miR-133b | hsa-miRNA-133b | 480871_mir |
| miR-206 | hsa-miRNA-206 | 477968_mir |
| miR-191-5p | hsa-miRNA-191-5p | 477952_mir |
| miR-16-5p | hsa-miRNA-16-5p | 477860_mir |
| *PGD* | Phosphogluconate dehydrogenase | Hs00427230_m1 |
| *G6PD* | Glucose-6-phosphate dehydrogenase | Hs00166169_m1 |
| *TKT* | Transketolase | Hs01115545_m1 |
| *HDAC4* | Histone deacetylase 4 | Hs01041648_m1 |
| *FGFR1* | Fibroblast growth factor receptor 1 | Hs00241111_m1 |
| *SP1* | Sp1 transcription factor | Hs00916521_m1 |
| *TGFB1* | Transforming growth factor beta 1 | Hs00998133_m1 |
| *KCNQ1* | Potassium voltage-gated channel subfamily Q member 1 | Hs00923522_m1 |
| *IGF1R* | Insulin-like growth factor 1 receptor | Hs00609566_m1 |
| *ANXA2* | Annexin A2 | Hs00743063_s1 |
| *SMN2* | Survival motor neuron 2 | Hs00165806_m1 |
| *ACTB* | Actin beta | Hs01060665_g1 |
| *RPLP0* | Ribosomal protein lateral stalk subunit P0 | Hs00420895_gH |

**Table S2**. Correlation analysis between the relative expression of selected RNAs, *SMN* transcripts, and the *SMN*-FL/*SMN*-Δ7 ratio, and age (n = 50).

| **RNA entity** | **Age (T0)** | |
| --- | --- | --- |
|  | **r** | ***p*-value** |
| miR-1-3p | -0.208 | 0.146* |
| miR-133a-3p | 0.053 | 0.716 |
| miR-133b | -0.081 | 0.577 |
| miR-206 | 0.033 | 0.821* |
| *PGD* [1] | 0.326 | **0.022** |
| *G6PD* [1] | 0.340 | **0.017*** |
| *TKT* [1] | 0.275 | 0.055 |
| *HDAC4* [1] | 0.244 | 0.091 |
| *FGFR1* [1] | 0.299 | **0.037** |
| *SP1* [1] | 0.182 | 0.211 |
| *TGFB1* [1] | 0.111 | 0.449 |
| *KCNQ1* [1] | 0.276 | 0.055 |
| *IGF1R* [1] | 0.180 | 0.216 |
| *ANXA2* [1] | 0.137 | 0.349 |
| *LINCMD1* [2] | -0.208 | 0.156 |
| *lnc-GJA1-2* [2] | 0.032 | 0.828 |
| *SMN*-total [1] | 0.055 | 0.708* |
| *SMN*-FL [1] | -0.037 | 0.801 |
| *SMN*-Δ7 [1] | 0.085 | 0.560 |
| *SMN*-FL/*SMN*-Δ7 [1] | -0.116 | 0.426* |

The number of missing data is indicated in [] brackets. Bold values in the table denote statistically significant differences. Pearson correlation was used for normally distributed data, and Spearman correlation* for non-normally distributed data. r, correlation coefficient

**Table S3.** Relative expression of selected RNAs and *SMN* transcripts, and the ratio of *SMN*-FL/*SMN*-Δ7 in male (n = 23) and female (n = 27) SMA patients.

| **RNA entity** | **Sex** | **Median (IQR)** | **Mean (SD)** | ***p*-value** |
| --- | --- | --- | --- | --- |
| miR-1-3p | Male | -7.433 (-8.005 – -6.986) | -7.374 (1.043) | 0.553* |
|  | Female | -7.355 (-8.000 – -6.928) | -7.269 (1.055) |  |
| miR-133a-3p | Male | -14.684 (-15.659 – -13.871) | -14.867 (1.185) | **0.014** |
|  | Female | -13.828 (-15.225 – -13.357) | -14.025 (1.144) |  |
| miR-133b | Male | -18.921 (-19.666 – -18.290) | -19.049 (1.151) | 0.134 |
|  | Female | -18.557 (-19.317 – -17.950) | -18.541 (1.202) |  |
| miR-206 | Male | -19.190 (-23.129 – -17.184) | -20.068 (3.586) | 0.823* |
|  | Female | -18.656 (-21.101 – -17.420) | -19.450 (2.630) |  |
| *PGD* [1] | Male | -3.757 (-4.298 – -3.536) | -3.870 (0.603) | 0.345 |
|  | Female | -4.027 (-4.431 – -3.578) | -4.016 (0.431) |  |
| *G6PD* [1] | Male | -4.273 (-4.488 – -4.115) | -4.217 (0.427) | 0.088* |
|  | Female | -4.459 (-4.742 – -4.203) | -4.429 (0.349) |  |
| *TKT* [1] | Male | -1.603 (-1.901 – -1.418) | -1.623 (0.468) | 0.531 |
|  | Female | -1.674 (-2.002 – -1.456) | -1.699 (0.356) |  |
| *HDAC4* [1] | Male | -6.821 (-7.041 – -6.510) | -6.804 (0.310) | 0.451 |
|  | Female | -6.951 (-7.072 – -6.642) | -6.873 (0.324) |  |
| *FGFR1* [1] | Male | -10.236 (-10.674 – -9.904) | -10.160 (0.721) | **0.007** |
|  | Female | -9.601 (-9.915 – -9.029) | -9.579 (0.718) |  |
| *SP1* [1] | Male | -5.662 (-5.828 – -5.542) | -5.584 (0.358) | 0.794* |
|  | Female | -5.620 (-5.940 – -5.343) | -5.638 (0.393) |  |
| *TGFB1* [1] | Male | -2.419 (-2.580 – -2.216) | -2.389 (0.264) | 0.696 |
|  | Female | -2.366 (-2.520 – -2.190) | -2.359 (0.258) |  |
| *KCNQ1* [1] | Male | -6.594 (-6.751 – -6.292) | -6.527 (0.387) | 0.112 |
|  | Female | -6.742 (-7.020 – -6.372) | -6.716 (0.429) |  |
| *IGF1R* [1] | Male | -4.729 (-4.962 – -4.402) | -4.640 (0.549) | 0.507 |
|  | Female | -4.811 (-5.214 – -4.240) | -4.749 (0.594) |  |
| *ANXA2* [1] | Male | -2.738 (-2.942 – -2.417) | -2.704 (0.322) | **0.032** |
|  | Female | -2.943 (-3.062 – -2.680) | -2.901 (0.292) |  |
| *LINCMD1* [2] | Male | -18.981 (-21.161 – -16.528) | -18.927 (2.507) | **0.047*** |
|  | Female | -20.380 (-21.975 – -18.621) | -20.473 (3.131) |  |
| *lnc-GJA1-2* [2] | Male | -8.668 (-9.338 – -8.063) | -8.759 (0.971) | 0.595 |
|  | Female | -8.749 (-9.142 – -8.063) | -8.607 (0.978) |  |
| *SMN*-total [1] | Male | -6.257 (-6.642 – -6.034 | -6.257 (0.363) | 0.536 |
|  | Female | -6.307 (-6.568 – -5.883) | -6.176 (0.538) |  |
| *SMN*-FL [1] | Male | -8.184 (-8.557 – -7.866) | -8.127 (0.454) | 0.610 |
|  | Female | -8.298 (-8.579 – -7.844) | -8.200 (0.549) |  |
| *SMN*-Δ7 [1] | Male | -8.409 (-8.681 – -8.231) | -8.402 (0.367) | 0.352 |
|  | Female | -8.516 (-8.896 – -8.265) | -8.526 (0.552) |  |
| *SMN*-FL/*SMN*-Δ7 [1] | Male | 0.315 (0.073 – 0.491) | 0.276 (0.209) | 0.560* |
|  | Female | 0.228 (0.098 – 0.379) | 0.326 (0.483) |  |

Data are presented as log_2_-transformed expression values. Bold values in the table denote statistically significant differences. The number of missing data is indicated in [] brackets. The Mann-Whitney U test* was used for non-normally distributed data, and the unpaired t-test was used for normally distributed data (Shapiro-Wilk test). SD, standard deviation; IQR, interquartile range.

**Table S4.** Relative expression of selected RNAs and *SMN* transcripts, and the ratio of *SMN*-FL/*SMN*-Δ7 between SMA patients with SMA type II (n = 22) and type III (n = 23).

| **RNA entity** | **SMA Type** | **Median (IQR)** | **Mean (SD)** | ***p*-value** |
| --- | --- | --- | --- | --- |
| miR-1-3p | II | -7.255 (-7.910 – -6.704) | -7.021 (1.230) | **0.048*** |
|  | III | -7.706 (-8.246 – -7.148) | -7.710 (0.621) |  |
| miR-133a-3p | II | -13.861 (-14.619 – -13.402) | -14.138 (1.150) | 0.148 |
|  | III | -14.743 (-15.596 – -13.536) | -14.666 (1.250) |  |
| miR-133b | II | -18.517 (-19.074 – -17.918) | -18.520 (1.349) | 0.173 |
|  | III | -18.922 (-19.666 – -18.290) | -19.030 (1.095) |  |
| miR-206 | II | -20.282 (-23.510 – -18.647) | -21.106 (2.976) | **0.004*** |
|  | III | -18.289 (-19.731 – -17.144) | -18.807 (2.691) |  |
| *PGD* [1] | II | -3.693 (-4.453 – -3.523) | -3.973 (0.578) | 0.707* |
|  | III | -3.862 (-4.072 – -3.711) | -3.870 (0.440) |  |
| *G6PD* [1] | II | -4.434 (-4.693 – -4.090) | -4.368 (0.383) | 0.622* |
|  | III [1] | -4.341 (-4.560 – -4.124) | -4.277 (0.433) |  |
| *TKT* [1] | II | -1.651 (-2.047 – -1.389) | -1.693 (0.411) | 0.944* |
|  | III [1] | -1.647 (-1.925 – -1.388) | -1.616 (0.426) |  |
| *HDAC4* [1] | II | -6.837 (-7.080 – -6.467) | -6.801 (0.355) | 0.576 |
|  | III [1] | -6.868 (-7.018 – -6.630) | -6.856 (0.290) |  |
| *FGFR1* [1] | II | -10.001 (-10.333 – -9.200) | -9.827 (0.831) | 0.760 |
|  | III [1] | -9.953 (-10.483 – -9.374) | -9.903 (0.802) |  |
| *SP1* [1] | II | -5.592 (-5.945 – -5.176) | -5.571 (0.411) | 0.610 |
|  | III [1] | -5.687 (-5.829 – -5.503) | -5.631 (0.358) |  |
| *TGFB1* [1] | II | -2.279 (-2.495 – -2.178) | -2.323 (0.271) | 0.221 |
|  | III [1] | -2.457 (-2.581 – -2.252) | -2.423 (0.258) |  |
| *KCNQ1* [1] | II | -6.752 (-7.081 – -6.319) | -6.718 (0.459) | 0.153 |
|  | III [1] | -6.551 (-6.807 – -6.280) | -6.528 (0.401) |  |
| *IGF1R* [1] | II | -4.861 (-5.279 – -4.168) | -4.740 (0.645) | 0.542* |
|  | III [1] | -4.729 (-5.040 – -4.495) | -4.637 (0.547) |  |
| *ANXA2* [1] | II | -2.901 (-3.149 – -2.705) | -2.860 (0.354) | 0.482 |
|  | III [1] | -2.817 (-3.034 – -2.597) | -2.790 (0.303) |  |
| *LINCMD1* [2] | II | -20.475 (-22.092 – -16.929) | -19.315 (3.247) | 0.903* |
|  | III [1] | -19.779 (-20.963 – -18.329) | -19.745 (2.146) |  |
| *lnc-GJA1-2* [2] | II | -8.562 (-9.191 – -7.599) | -8.441 (1.101) | 0.205 |
|  | III [1] | -8.863 (-9.343 – -8.354) | -8.833 (0.868) |  |
| *SMN*-total [1] | II | -6.447 (-6.655 – -5.908) | -6.304 (0.472) | 0.260* |
|  | III [1] | -6.197 (-6.536 – -5.929) | -6.132 (0.490) |  |
| *SMN*-FL [1] | II | -8.317 (-8.582 – -7.946) | -8.224 (0.457) | 0.635 |
|  | III [1] | -8.217 (-8.579 – -7.803) | -8.150 (0.561) |  |
| *SMN*-Δ7 [1] | II | -8.632 (-8.945 – -8.346) | -8.627 (0.421) | **0.040** |
|  | III [1] | -8.409 (-8.562 – -8.231) | -8.335 (0.490) |  |
| *SMN*-FL/*SMN*-Δ7 [1] | II | 0.325 (0.142 – 0.419) | 0.403 (0.510) | 0.082* |
|  | III [1] | 0.194 (0.044 – 0.332) | 0.184 (0.184) |  |

Data are presented as log_2_-transformed expression values. Bold values in the table denote statistically significant differences. The number of missing data is indicated in [] brackets. The Mann-Whitney U test* was used for non-normally distributed data, and the unpaired t-test was used for normally distributed data (Shapiro-Wilk test). SD, standard deviation; IQR, interquartile range.

**Table S5.** ANCOVA of baseline RNA expression (selected RNAs, *SMN* transcripts, and the *SMN*-FL/*SMN*-Δ7 ratio) comparing SMA type II (n = 19) versus III (n = 21) patients, adjusted for *SMN2* copy number.

| **RNA entity** | **Effect** | **df1** | **df2** | **F** | ***p*_adj_-value** |
| --- | --- | --- | --- | --- | --- |
| miR-1-3p | *SMN2* CN | 1 | 37 | 0.009 | 0.062 |
|  | SMA Type | 1 |  | 3.701 |  |
| miR-133a-3p | *SMN2* CN | 1 | 37 | 1.229 | 0.238 |
|  | SMA Type | 1 |  | 1.440 |  |
| miR-133b | *SMN2* CN | 1 | 37 | 0.094 | 0.148 |
|  | SMA Type | 1 |  | 2.186 |  |
| miR-206 | *SMN2* CN | 1 | 37 | 0.952 | 0.185 |
|  | SMA Type | 1 |  | 1.826 |  |
| *PGD* [1] | *SMN2* CN | 1 | 36 | 0.184 | 0.836 |
|  | SMA Type | 1 |  | 0.044 |  |
| *G6PD* [1] | *SMN2* CN | 1 | 36 | 0.072 | 0.526 |
|  | SMA Type | 1 |  | 0.410 |  |
| *TKT* [1] | *SMN2* CN | 1 | 36 | 0.115 | 0.785 |
|  | SMA Type | 1 |  | 0.075 |  |
| *HDAC4* [1] | *SMN2* CN | 1 | 36 | 0.134 | 0.903 |
|  | SMA Type | 1 |  | 0.015 |  |
| *FGFR1* [1] | *SMN2* CN | 1 | 36 | 0.754 | 0.769 |
|  | SMA Type | 1 |  | 0.088 |  |
| *SP1* [1] | *SMN2* CN | 1 | 36 | 0.319 | 0.941 |
|  | SMA Type | 1 |  | 0.006 |  |
| *TGFB1* [1] | *SMN2* CN | 1 | 36 | 0.073 | 0.512 |
|  | SMA Type | 1 |  | 0.438 |  |
| *KCNQ1* [1] | *SMN2* CN | 1 | 36 | 0.329 | 0.422 |
|  | SMA Type | 1 |  | 0.660 |  |
| *IGF1R* [1] | *SMN2* CN | 1 | 36 | 0.147 | 0.418 |
|  | SMA Type | 1 |  | 0.670 |  |
| *ANXA2* [1] | *SMN2* CN | 1 | 36 | 0.182 | 0.715 |
|  | SMA Type | 1 |  | 0.135 |  |
| *LINCMD1* [2] | *SMN2* CN | 1 | 35 | 0.095 | 0.506 |
|  | SMA Type | 1 |  | 0.451 |  |
| *lnc-GJA1-2* [2] | *SMN2* CN | 1 | 35 | 0.843 | 0.146 |
|  | SMA Type | 1 |  | 2.206 |  |
| *SMN*-total [1] | *SMN2* CN | 1 | 36 | 3.613 | 0.986 |
|  | SMA Type | 1 |  | 0.000 |  |
| *SMN*-FL [1] | *SMN2* CN | 1 | 36 | 7.054 | 0.328 |
|  | SMA Type | 1 |  | 0.985 |  |
| *SMN*-Δ7 [1] | *SMN2* CN | 1 | 36 | 2.174 | 0.255 |
|  | SMA Type | 1 |  | 1.339 |  |
| *SMN*-FL/*SMN*-Δ7 [1] | *SMN2* CN | 1 | 36 | 1.991 | **0.038** |
|  | SMA Type | 1 |  | 4.665 |  |

For SMA type *SMN2* copy number effects, df1 = 1; df2 is the model error df for each RNA. Bold values in the table denote statistically significant differences. The number of missing data is indicated in [] brackets. Adj, covariate-adjusted model; df, degrees of freedom; F, F statistics; CN, copy number.

**Table S6.** Relative expression of selected RNAs and *SMN* transcripts, and the ratio of *SMN*-FL/*SMN*-Δ7 between ambulatory (n = 13) and non-ambulatory (n = 37) SMA patients.

| **RNA entity** | **Ambulatory status** | **Median (IQR)** | **Mean (SD)** | ***p*-value** |
| --- | --- | --- | --- | --- |
| miR-1-3p | Non-ambulatory | -7.427 (−7.963 – -6.913) | -7.258 (1.039) | 0.370* |
|  | Ambulatory | -7.706 (−8.288 – -6.963) | -7.487 (1.067) |  |
| miR-133a-3p | Non-ambulatory | -14.027 (−14.970 – -13.392) | -14.229 (1.198) | 0.082 |
|  | Ambulatory | -15.225 (−15.714 – -13.898) | -14.936 (1.200) |  |
| miR-133b | Non-ambulatory | -18.557 (−19.171 – -18.070) | -18.583 (1.158) | 0.064 |
|  | Ambulatory | -19.158 (−19.889 – -18.480) | -19.319 (1.170) |  |
| miR-206 | Non-ambulatory | -19.731 (−22.085 – -18.436) | -20.492 (2.779) | **< 0.001*** |
|  | Ambulatory | -17.144 (−17.562 – -16.243) | -17.683 (3.066) |  |
| *PGD* [1] | Non-ambulatory | -3.755 (−4.168 – -3.560) | -3.905 (0.491) | 0.094* |
|  | Ambulatory | -4.161 (−4.548 – -3.813) | -4.090 (0.582) |  |
| *G6PD* [1] | Non-ambulatory | -4.378 (−4.565 – -4.121) | -4.333 (0.367) | 0.693* |
|  | Ambulatory | -4.445 (−4.729 – -4.117) | -4.335 (0.495) |  |
| *TKT* [1] | Non-ambulatory | -1.674 (−1.979 – -1.438) | -1.681 (0.377) | 0.694 |
|  | Ambulatory | -1.611 (−1.917 – -1.329) | -1.617 (0.505) |  |
| *HDAC4* [1] | Non-ambulatory | -6.834 (−7.052 – -6.495) | -6.800 (0.332) | 0.053 |
|  | Ambulatory | -6.992 (−7.150 – -6.778) | -6.972 (0.226) |  |
| *FGFR1* [1] | Non-ambulatory | -9.991 (−10.303 – -9.433) | -9.857 (0.743) | 0.365* |
|  | Ambulatory | -9.514 (−10.445 – -9.131) | -9.785 (0.879) |  |
| *SP1* [1] | Non-ambulatory | -5.650 (−5.839 – -5.404) | -5.615 (0.365) | 0.968 |
|  | Ambulatory | -5.657 (−5.947 – -5.278) | -5.609 (0.420) |  |
| *TGFB1* [1] | Non-ambulatory | -2.338 (−2.529 – -2.192) | -2.369 (0.255) | 0.886 |
|  | Ambulatory | -2.451 (−2.610 – -2.183) | -2.382 (0.279) |  |
| *KCNQ1* [1] | Non-ambulatory | -6.570 (−6.912 – -6.311) | -6.625 (0.457) | 0.831 |
|  | Ambulatory | -6.675 (−6.823 – -6.434) | -6.649 (0.277) |  |
| *IGF1R* [1] | Non-ambulatory | -4.755 (−5.156 – -4.286) | -4.678 (0.585) | 0.633 |
|  | Ambulatory | -4.838 (−5.147 – -4.551) | -4.768 (0.547) |  |
| *ANXA2* [1] | Non-ambulatory | -2.871 (−3.054 – -2.556) | -2.800 (0.351) | 0.511 |
|  | Ambulatory | -2.925 (−3.024 – -2.692) | -2.852 (0.189) |  |
| *LINCMD1* [2] | Non-ambulatory | -20.380 (−21.836 – -17.958) | -19.727 (2.928) | 0.584* |
|  | Ambulatory | -19.169 (−19.980 – -18.396) | -19.878 (3.096) |  |
| *lnc-GJA1-2* [2] | Non-ambulatory | -8.683 (−9.328 – -7.980) | -8.668 (1.036) | 0.868 |
|  | Ambulatory | -8.799 (−9.319 – -8.138) | -8.712 (0.761) |  |
| *SMN*-total [1] | Non-ambulatory | -6.395 (−6.643 – -6.061) | -6.333 (0.393) | **0.006** |
|  | Ambulatory | -5.913 (−6.174 – -5.597) | -5.841 (0.489) |  |
| *SMN*-FL [1] | Non-ambulatory | -8.335 (−8.585 – -8.009) | -8.298 (0.428) | **0.006** |
|  | Ambulatory | -7.805 (−8.158 – -7.382) | - 7.764 (0.526) |  |
| *SMN*-Δ7 [1] | Non-ambulatory | -8.542 (−8.887 – -8.335) | -8.608 (0.386) | **0.003** |
|  | Ambulatory | -8.210 (−8.382 – -7.758) | -8.049 (0.501) |  |
| *SMN*-FL/*SMN*-Δ7 [1] | Non-ambulatory | 0.228 (0.075 – 0.387) | 0.310 (0.431) | 0.500* |
|  | Ambulatory | 0.314 (0.133 – 0.418) | 0.284 (0.170) |  |

Data are presented as log_2_-transformed expression values. Bold values in the table denote statistically significant differences. The number of missing data is indicated in [] brackets. The Mann-Whitney U test* was used for non-normally distributed data, and the unpaired t-test was used for normally distributed data (Shapiro-Wilk test). SD, standard deviation; IQR, interquartile range.

**Table S7.** ANCOVA of baseline RNA expression (selected RNAs, *SMN* transcripts, and the *SMN*-FL/*SMN*-Δ7 ratio) comparing ambulatory (n = 11) versus non-ambulatory (n = 31) SMA patients, adjusted for age, disease duration and *SMN2* copy number.

| **RNA entity** | **Effect** | **df1** | **df2** | **F** | ***p*_adj_-value** |
| --- | --- | --- | --- | --- | --- |
| miR-1-3p | Age | 1 | 37 | 0.772 | 0.855 |
|  | Disease duration | 1 |  | 0.074 |  |
|  | *SMN2* CN | 1 |  | 0.075 |  |
|  | Ambulatory status | 1 |  | 0.034 |  |
| miR-133a-3p | Age | 1 | 37 | 0.423 | 0.118 |
|  | Disease duration | 1 |  | 0.013 |  |
|  | *SMN2* CN | 1 |  | 1.123 |  |
|  | Ambulatory status | 1 |  | 2.568 |  |
| miR-133b | Age | 1 | 37 | 0.341 | 0.096 |
|  | Disease duration | 1 |  | 0.235 |  |
|  | *SMN2* CN | 1 |  | 0.145 |  |
|  | Ambulatory status | 1 |  | 2.920 |  |
| miR-206 | Age | 1 | 37 | 4.733 | **0.032** |
|  | Disease duration | 1 |  | 3.495 |  |
|  | *SMN2* CN | 1 |  | 5.306 |  |
|  | Ambulatory status | 1 |  | 4.954 |  |
| *PGD* [1] | Age | 1 | 36 | 0.749 | 0.426 |
|  | Disease duration | 1 |  | 0.933 |  |
|  | *SMN2* CN | 1 |  | 0.008 |  |
|  | Ambulatory status | 1 |  | 0.648 |  |
| *G6PD* [1] | Age | 1 | 36 | 1.104 | 0.824 |
|  | Disease duration | 1 |  | 0.077 |  |
|  | *SMN2* CN | 1 |  | 0.015 |  |
|  | Ambulatory status | 1 |  | 0.050 |  |
| *TKT* [1] | Age | 1 | 36 | 0.020 | 0.512 |
|  | Disease duration | 1 |  | 1.759 |  |
|  | *SMN2* CN | 1 |  | 0.001 |  |
|  | Ambulatory status | 1 |  | 0.439 |  |
| *HDAC4* [1] | Age | 1 | 36 | 0.778 | 0.279 |
|  | Disease duration | 1 |  | 1.171 |  |
|  | *SMN2* CN | 1 |  | 0.099 |  |
|  | Ambulatory status | 1 |  | 1.208 |  |
| *FGFR1* [1] | Age | 1 | 36 | 0.122 | 0.421 |
|  | Disease duration | 1 |  | 4.590 |  |
|  | *SMN2* CN | 1 |  | 0.317 |  |
|  | Ambulatory status | 1 |  | 0.662 |  |
| *SP1* [1] | Age | 1 | 36 | 0.028 | 0.395 |
|  | Disease duration | 1 |  | 1.040 |  |
|  | *SMN2* CN | 1 |  | 0.781 |  |
|  | Ambulatory status | 1 |  | 0.741 |  |
| *TGFB1* [1] | Age | 1 | 36 | 0.310 | 0.840 |
|  | Disease duration | 1 |  | 0.168 |  |
|  | *SMN2* CN | 1 |  | 0.514 |  |
|  | Ambulatory status | 1 |  | 0.041 |  |
| *KCNQ1* [1] | Age | 1 | 36 | 0.174 | 0.715 |
|  | Disease duration | 1 |  | 0.956 |  |
|  | *SMN2* CN | 1 |  | 1.168 |  |
|  | Ambulatory status | 1 |  | 0.136 |  |
| *IGF1R* [1] | Age | 1 | 36 | 0.004 | 0.684 |
|  | Disease duration | 1 |  | 1.322 |  |
|  | *SMN2* CN | 1 |  | 0.171 |  |
|  | Ambulatory status | 1 |  | 0.169 |  |
| *ANXA2* [1] | Age | 1 | 36 | 0.308 | 0.307 |
|  | Disease duration | 1 |  | 0.056 |  |
|  | *SMN2* CN | 1 |  | 1.340 |  |
|  | Ambulatory status | 1 |  | 1.072 |  |
| *LINCMD1* [2] | Age | 1 | 35 | 4.692 | 0.337 |
|  | Disease duration | 1 |  | 1.023 |  |
|  | *SMN2* CN | 1 |  | 0.064 |  |
|  | Ambulatory status | 1 |  | 0.948 |  |
| *lnc-GJA1-2* [2] | Age | 1 | 35 | 0.009 | 0.969 |
|  | Disease duration | 1 |  | 0.000 |  |
|  | *SMN2* CN | 1 |  | 0.024 |  |
|  | Ambulatory status | 1 |  | 0.002 |  |
| *SMN*-total [1] | Age | 1 | 36 | 1.432 | **0.016** |
|  | Disease duration | 1 |  | 1.405 |  |
|  | *SMN2* CN | 1 |  | 2.004 |  |
|  | Ambulatory status | 1 |  | 6.410 |  |
| *SMN*-FL [1] | Age | 1 | 36 | 5.731 | **0.008** |
|  | Disease duration | 1 |  | 1.705 |  |
|  | *SMN2* CN | 1 |  | 5.040 |  |
|  | Ambulatory status | 1 |  | 7.958 |  |
| *SMN*-Δ7 [1] | Age | 1 | 36 | 2.200 | **0.023** |
|  | Disease duration | 1 |  | 0.797 |  |
|  | *SMN2* CN | 1 |  | 5.624 |  |
|  | Ambulatory status | 1 |  | 5.655 |  |
| *SMN*-FL/*SMN*-Δ7 [1] | Age | 1 | 36 | 1.023 | 0.563 |
|  | Disease duration | 1 |  | 0.222 |  |
|  | *SMN2* CN | 1 |  | 0.000 |  |
|  | Ambulatory status | 1 |  | 0.341 |  |

For age, disease duration, *SMN2* copy number and ambulatory status effects, df1 = 1. df2 is the model error df for each RNA. Bold values in the table denote statistically significant differences. The number of missing data is indicated in [] brackets. Adj, covariate-adjusted model; df, degrees of freedom; F, F statistics; CN, copy number.

**Table S8**. Correlation analysis between the relative expression of selected RNAs, *SMN* transcripts, and the *SMN*-FL/*SMN*-Δ7 ratio, and clinical outcome measures including RHS score (n = 49), RULM score (n = 48), VC (%, n = 49), and PEF (%, n = 49).

| **RNA entity** | **RHS** | | **RULM** | | **VC (%)** | | **PEF (%)** | |
| --- | --- | --- | --- | --- | --- | --- | --- | --- |
|  | **r** | ***p*-value** | **r** | ***p*-value** | **r** | ***p*-value** | **r** | ***p*-value** |
| miR-1-3p | -0.207 | 0.154* | -0.152 | 0.301* | -0.245 | 0.090* | -0.255 | 0.120* |
| miR-133a-3p | -0.443 | **0.001*** | -0.450 | **0.001** | -0.471 | **<0.001** | -0.448 | **0.001** |
| miR-133b | -0.449 | **0.001*** | -0.325 | **0.024** | -0.393 | **0.005** | -0.376 | **0.008** |
| miR-206 | 0.656 | **<0.001*** | 0.459 | **0.001*** | 0.543 | **<0.001*** | 0.467 | **<0.001*** |
| *PGD* [1] | -0.236 | 0.107* | -0.161 | 0.279 | 0.102 | 0.491 | -0.008 | 0.955 |
| *G6PD* [1] | -0.133 | 0.367* | 0.132 | 0.417* | 0.054 | 0.714* | -0.012 | 0.936* |
| *TKT* [1] | -0.143 | 0.333* | -0.104 | 0.485 | 0.062 | 0.673 | 0.010 | 0.944 |
| *HDAC4* [1] | -0.314 | **0.030*** | -0.286 | 0.051 | -0.112 | 0.449 | -0.180 | 0.222 |
| *FGFR1* [1] | 0.002 | 0.987* | -0.027 | 0.858 | -0.009 | 0.952 | -0.007 | 0.960 |
| *SP1* [1] | -0.042 | 0.779* | 0.060 | 0.690 | 0.145 | 0.324 | 0.047 | 0.753 |
| *TGFB1* [1] | -0.064 | 0.667* | -0.031 | 0.837 | 0.057 | 0.700 | 0.028 | 0.853 |
| *KCNQ1* [1] | -0.153 | 0.300* | -0.078 | 0.602 | -0.012 | 0.937 | -0.131 | 0.373 |
| *IGF1R* [1] | -0.223 | 0.128* | -0.157 | 0.291 | -0.043 | 0.772 | -0.118 | 0.423 |
| *ANXA2* [1] | 0.037 | 0.801* | -0.112 | 0.454 | 0.001 | 0.994 | 0.037 | 0.804 |
| *LINCMD1* [2] | 0.317 | **0.030*** | 0.164 | 0.320 | 0.089 | 0.550 | 0.153 | 0.303 |
| *lnc-GJA1-2* [2] | -0.107 | 0.473* | 0.027 | 0.869 | -0.006 | 0.970 | -0.078 | 0.604 |
| *SMN*-total [1] | 0.408 | **0.004*** | 0.294 | **0.045*** | 0.318 | **0.028*** | 0.295 | **0.042*** |
| *SMN*-FL [1] | 0.494 | **< 0.001*** | 0.379 | **0.009** | 0.370 | **0.010** | 0.423 | **0.003** |
| *SMN*-Δ7 [1] | 0.637 | **< 0.001*** | 0.502 | **< 0.001** | 0.506 | **< 0.001** | 0.515 | **< 0.001** |
| *SMN*-FL/*SMN*-Δ7  [1] | -0.016 | 0.913* | -0.014 | 0.926* | 0.015 | 0.919* | 0.076 | 0.610* |

The number of missing data is indicated in [] brackets. Bold values in the table denote statistically significant differences. Pearson correlation was used for normally distributed data, and Spearman correlation* for non-normally distributed data. r, correlation coefficient; RHS, Revised Hammersmith Scale; RULM, Revised Upper Limb Module; VC (%), Vital Capacity; PEF (%), Peak Expiratory Flow.

**Table S9**. Correlation analysis between the relative expression of selected RNAs, *SMN* transcripts, and the *SMN*-FL/*SMN*-Δ7 ratio, and clinical outcome measures including RHS score (n = 37), RULM score (n = 36), VC (%, n = 37), and PEF (%, n = 37), adjusted for age, disease duration, and *SMN2* copy number.

| **RNA entity** | **RHS** | | **RULM** | | **VC (%)** | | **PEF (%)** | |
| --- | --- | --- | --- | --- | --- | --- | --- | --- |
|  | **r** | ***p*_adj_-value** | **r** | ***p*_adj_-value** | **r** | ***p*_adj_-value** | **r** | ***p*_adj_-value** |
| miR-1-3p | 0.059 | 0.721 | -0.072 | 0.668 | -0.107 | 0.518 | -0.038 | 0.820 |
| miR-133a-3p | -0.215 | 0.189 | -0.280 | 0.089 | -0.430 | **0.006** | -0.340 | **0.034** |
| miR-133b | -0.192 | 0.241 | -0.267 | 0.105 | -0.379 | **0.017** | -0.341 | **0.033** |
| miR-206 | 0.430 | **0.006** | 0.389 | **0.016** | 0.428 | **0.007** | 0.346 | **0.031** |
| *PGD* [1] | -0.217 | 0.190 | -0.128 | 0.452 | 0.089 | 0.596 | -0.048 | 0.775 |
| *G6PD* [1] | -0.118 | 0.479 | -0.145 | 0.393 | 0.148 | 0.375 | 0.030 | 0.858 |
| *TKT* [1] | -0.040 | 0.810 | -0.081 | 0.636 | 0.004 | 0.982 | -0.043 | 0.799 |
| *HDAC4* [1] | -0.233 | 0.158 | -0.290 | 0.081 | -0.124 | 0.458 | -0.189 | 0.257 |
| *FGFR1* [1] | 0.239 | 0.148 | 0.166 | 0.325 | -0.030 | 0.860 | 0.075 | 0.654 |
| *SP1* [1] | 0.095 | 0.569 | 0.212 | 0.207 | 0.276 | 0.094 | 0.144 | 0.389 |
| *TGFB1* [1] | -0.008 | 0.960 | 0.059 | 0.730 | 0.184 | 0.268 | 0.117 | 0.486 |
| *KCNQ1* [1] | -0.102 | 0.543 | -0.136 | 0.421 | -0.170 | 0.306 | -0.255 | 0.122 |
| *IGF1R* [1] | 0.012 | 0.943 | -0.020 | 0.907 | 0.069 | 0.682 | -0.014 | 0.934 |
| *ANXA2* [1] | -0.188 | 0.259 | -0.321 | 0.053 | -0.214 | 0.197 | -0.102 | 0.541 |
| *LINCMD1* [2] | 0.201 | 0.233 | 0.285 | 0.092 | 0.154 | 0.363 | 0.202 | 0.230 |
| *lnc-GJA1-2* [2] | -0.062 | 0.717 | -0.012 | 0.943 | 0.060 | 0.723 | -0.062 | 0.714 |
| *SMN*-total [1] | 0.347 | **0.033** | 0.265 | 0.113 | 0.251 | 0.128 | 0.297 | 0.070 |
| *SMN*-FL [1] | 0.442 | **0.005** | 0247 | 0.140 | 0.297 | 0.070 | 0.396 | **0.014** |
| *SMN*-Δ7 [1] | 0.398 | **0.013** | 0.339 | **0.040** | 0.293 | 0.074 | 0.323 | **0.048** |
| *SMN*-FL/*SMN*-Δ7  [1] | 0.085 | 0.610 | -0.074 | 0.662 | 0.030 | 0.856 | 0.111 | 0.507 |

The number of missing data is indicated in [] brackets. Bold values in the table denote statistically significant differences. For adjusted analyses, values are partial Pearson correlation coefficients, controlling for age, disease duration, and *SMN2* copy number. Adj, adjusted model; r, correlation coefficient; RHS, Revised Hammersmith Scale; RULM, Revised Upper Limb Module; VC (%), Vital Capacity; PEF (%), Peak Expiratory Flow.

**Table S10**. Correlation analysis between the relative expression of selected RNAs, *SMN* transcripts, and the *SMN*-FL/*SMN*-Δ7 ratio, and clinical outcome measures including RHS score and RULM score with the exclusion of ceiling and floor values.

| **RNA entity** | **RHS (n = 38)** | | **RULM (n = 41)** | | **RHS (n = 29)** | | **RULM (n = 30)** | |
| --- | --- | --- | --- | --- | --- | --- | --- | --- |
|  | **r** | ***p*-value** | **r** | ***p*-value** | **r** | ***p*_adj_-value** | **r** | ***p*_adj_-value** |
| miR-1-3p | 0.045 | 0.790* | -0.254 | 0.110* | 0.162 | 0.385 | -0.246 | 0.174 |
| miR-133a-3p | -0.273 | 0.098* | -0.455 | **0.003** | -0.136 | 0.466 | -0.379 | **0.032** |
| miR-133b | -0.204 | 0.219* | -0.404 | **0.009** | -0.113 | 0.545 | -0.455 | **0.009** |
| miR-206 | 0.643 | **<0.001*** | 0.315 | **0.045*** | 0.431 | **0.015** | 0.247 | 0.172 |
| *PGD* [1] | -0.084 | 0.620* | 0.092 | 0.570 | -0.286 | 0.125 | 0.065 | 0.728 |
| *G6PD* [1] | -0.129 | 0.445* | 0.132 | 0.417* | -0.191 | 0.313 | 0.046 | 0.806 |
| *TKT* [1] | 0.096 | 0.572* | 0.130 | 0.425 | -0.033 | 0.863 | 0.082 | 0.661 |
| *HDAC4* [1] | -0.087 | 0.609* | -0.163 | 0.314 | -0.233 | 0.216 | -0.277 | 0.131 |
| *FGFR1* [1] | 0.260 | 0.120* | -0.046 | 0.777 | 0.346 | 0.061 | -0.081 | 0.665 |
| *SP1* [1] | -0.025 | 0.885* | 0.232 | 0.149 | 0.027 | 0.887 | 0.271 | 0.141 |
| *TGFB1* [1] | 0.062 | 0.717* | 0.202 | 0.211 | -0.055 | 0.773 | 0.226 | 0.222 |
| *KCNQ1* [1] | 0.176 | 0.297* | -0.068 | 0.677 | -0.084 | 0.660 | -0.150 | 0.419 |
| *IGF1R* [1] | 0.044 | 0.794* | -0.069 | 0.671 | -0.030 | 0.873 | -0.020 | 0.915 |
| *ANXA2* [1] | 0.046 | 0.787* | -0.089 | 0.583 | -0.220 | 0.242 | -0.236 | 0.202 |
| *LINCMD1* [2] | 0.137 | 0.427* | 0.164 | 0.320 | 0.184 | 0.341 | 0.122 | 0.522 |
| *lnc-GJA1-2* [2] | -0.144 | 0.403* | 0.027 | 0.869 | -0.057 | 0.769 | 0.020 | 0.917 |
| *SMN*-total [1] | 0.576 | **< 0.001*** | 0.212 | 0.189***** | 0.376 | **0.040** | 0.114 | 0.543 |
| *SMN*-FL [1] | 0.538 | **< 0.001*** | 0.253 | 0.115 | 0.413 | **0.023** | 0.022 | 0.908 |
| *SMN*-Δ7 [1] | 0.596 | **< 0.001*** | 0.298 | 0.062 | 0.397 | **0.030** | 0.156 | 0.403 |
| *SMN*-FL/*SMN*-Δ7  [1] | 0.169 | 0.317* | -0.008 | 0.959* | 0.070 | 0.714 | -0.128 | 0.491 |

The number of missing data is indicated in [] brackets. Bold values in the table denote statistically significant differences. In unadjusted analyses, Pearson correlation was used for normally distributed data, and Spearman correlation* for non-normally distributed data. For adjusted analyses, values are partial Pearson correlation coefficients, controlling for age, disease duration, and *SMN2* copy number. Adj, adjusted model; r, correlation coefficient; RHS, Revised Hammersmith Scale; RULM, Revised Upper Limb Module; VC (%), Vital Capacity; PEF (%), Peak Expiratory Flow.

**Table S11.** Relative expression of selected RNAs and *SMN* transcripts, and the log_2_(*SMN*-FL/*SMN*-Δ7) between SMA patients with 3 (n = 24) and 4 (n = 17) *SMN2* copies.

| **RNA entity** | ***SMN2* copy number** | **Median (IQR)** | **Mean (SD)** | ***p*-value** |
| --- | --- | --- | --- | --- |
| *SMN*-total | 3 | -6.418 (-6.671 – -5.998) | -6.372 (0.386) | **0.017** |
|  | 4 | -6.103 (-6.412 – -5.668) | -6.038 (0.438) |  |
| *SMN*-FL | 3 | -8.371 (-8.588 – -8.137) | -8.344 (0.341) | **0.005** |
|  | 4 | -7.899 (-8.306 – -7.426) | -7.909 (0.508) |  |
| *SMN*-Δ7 | 3 | -8.523 (-8.869 – -8.364) | -8.617 (0.356) | **0.005** |
|  | 4 | -8.245 (-8.568 – -7.809) | -8.247 (0.409) |  |
| *SMN*-FL/*SMN*-Δ7 | 3 | 0.207 (0.086 – 0.322) | 0.272 (0.441) | 0.236* |
|  | 4 | 0.340 (0.044 – 0.458) | 0.338 (0.371) |  |

Data are presented as log_2_-transformed expression values. Bold values in the table denote statistically significant differences. The Mann-Whitney U test* was used for non-normally distributed data, and the unpaired t-test was used for normally distributed data (Shapiro-Wilk test). SD, standard deviation; IQR, interquartile range.

**Table S12.** Log_2_ fold-change for selected RNAs and *SMN* transcripts, and Δlog_2_(*SMN*-FL/*SMN*-Δ7), in patients with SMA treated with nusinersen at T24 (n = 24).

| **RNA entity** | **Median (IQR) at T24** | **Mean (SD) at T24** | ***p*-value (T0 vs T24)** |
| --- | --- | --- | --- |
| miR-1-3p | -0.282 (-0.666 – 0.308) | -0.187 (0.965) | 0.353 |
| miR-133a-3p | 0.112 (-0.304 – 0.533) | -0.031 (0.822) | 0.857 |
| miR-133b | -0.046 (-0.920 – 0.450) | -0.130 (1.164) | 0.407* |
| miR-206 | -1.962 (-6.362 – 0.134) | -2.463 (4.432) | **0.012** |
| *PGD* | -0.345 (-0.534 – 0.060) | -0.289 (0.445) | **0.004** |
| *G6PD* | -0.159 (-0.485 – 0.030) | -0.257 (0.338) | **0.001** |
| *TKT* | -0.229 (-0.617 – 0.071) | -0.309 (0.406) | **0.001** |
| *HDAC4* | -0.163 (-0.458 – 0.109) | -0.158 (0.329) | **0.027** |
| *FGFR1* | -0.008 (-0.312 – 0.277) | -0.011 (0.341) | 0.878 |
| *SP1* | -0.157 (-0.434 – -0.015) | -0.268 (0.394) | **0.003** |
| *TGFB1* | -0.102 (-0.304 – -0.003) | -0.141 (0.211) | **0.003** |
| *KCNQ1* | -0.230 (-0.432 – -0.020) | -0.232 (0.274) | **< 0.001** |
| *IGF1R* | -0.347 (-0.745 – -0.010) | -0.371 (0.443) | **< 0.001** |
| *ANXA2* | 0.125 (-0.245 – 0.297) | 0.042 (0.318) | 0.527 |
| *LINCMD1* | -2.915 (-5.378 – -1.025) | -2.697 (3.662) | **0.001** |
| *lnc-GJA1-2* [1] | -0.866 (-1.316 – -0.87) | -0.768 (0.627) | **< 0.001** |
| *SMN*-total | -0.231 (-0.477 – -0.005) | -0.198 (0.374) | **0.016** |
| *SMN*-FL | -0.154 (-0.351 – 0.101) | -0.066 (0.394) | 0.179* |
| *SMN*-Δ7 | -0.147 (-0.400 – 0.157) | -0.144 (0.321) | **0.039** |
| *SMN*-FL/*SMN*-Δ7 | 0.017 (-0.065 – 0.256) | 0.078 (0.294) | 0.207 |

Bold values in the table denote statistically significant differences. One-sample t-test was used for normally distributed data, or the Wilcoxon signed-rank test* for non-normally distributed data. Time points T24 for each patient were normalized to baseline samples (T0) and obtained using the 2^-ΔΔCt^ method. SD, standard deviation; IQR, interquartile range.

**Table S13.** Log_2_ fold change for selected RNAs and *SMN* transcripts, and Δlog_2_(*SMN*-FL/*SMN*-Δ7), in patients with SMA treated with risdiplam at T6 (n = 12).

| **RNA entity** | **Median (IQR) at T6** | **Mean (SD) at T6** | ***p*-value (T0 vs T6)** |
| --- | --- | --- | --- |
| miR-1-3p | 0.126 (-0.953 – 0.402) | -0.288 (1.194) | 0.411 |
| miR-133a-3p | -0.646 (-1.167 – -0.171) | -0.680 (0.687) | **0.006** |
| miR-133b | -0.435 (-0.912 – 0.989) | 0.012 (1.475) | 0.978 |
| miR-206 | -0.340 (-1.646 – 2.401) | 0.053 (4.104) | 0.965 |
| *PGD* | 0.070 (-0.679 – 0.577) | 0.265 (1.197) | 0.754* |
| *G6PD* | 0.061 (-0.353 – 0.476) | 0.256 (1.074) | 0.638* |
| *TKT* | -0.045 (-0.505 – 0.534) | 0.183 (1.138) | 0.814* |
| *HDAC4* | 0.074 (-0.425 – 0.481) | 0.247 (1.054) | 0.814* |
| *FGFR1* | -0.057 (-0.347 – 0.263) | 0.078 (0.774) | 0.937* |
| *SP1* | 0.007 (-0.250 – 0.405) | 0.231 (1.082) | 0.754* |
| *TGFB1* | 0.081 (-0.254 – 0.269) | 0.230 (0.871) | 0.754* |
| *KCNQ1* | -0.185 (-0.270 – 0.380) | 0.192 (0.945) | 0.937* |
| *IGF1R* | 0.126 (-0.488 – 0.556) | 0.307 (1.296) | 0.583* |
| *ANXA2* | -0.068 (-0.274 – 0.411) | 0.191 (0.703) | 0.583* |
| *LINCMD1* [2] | 0.965 (-2.085 – 2.921) | 0.439 (3.214) | 0.676 |
| *lnc-GJA1-2* | -0.288 (-1.074 – 1.263) | -0.137 (1.680) | 0.783 |
| *SMN*-total | -0.173 (-0.286 – 0.313) | -0.112 (0.435) | 0.393 |
| *SMN*-FL | 0.707 (0.389 – 0.941) | 0.722 (0.340) | **< 0.001** |
| *SMN*-Δ7 | -0.701 (-0.832 – -0.501) | -0.720 (0.374) | **< 0.001** |
| *SMN*-FL/*SMN*-Δ7 | 1.450 (1.048 – 1.715) | 1.443 (0.425) | **< 0.001** |

The number of missing data is indicated in [] brackets. Bold values in the table denote statistically significant differences. One-sample t-test was used for normally distributed data, or the Wilcoxon signed-rank test* for non-normally distributed data. Time points T6 and T12 for each patient were normalized to baseline samples (T0) and obtained using the 2^-ΔΔCt^ method. SD, standard deviation; IQR, interquartile range.

**Table S14.** Log_2_ fold change for selected RNAs and *SMN* transcripts, and Δlog_2_(*SMN*-FL/*SMN*-Δ7), in patients with SMA treated with risdiplam at T12 (n = 10).

| **RNA entity** | **Median (IQR) at T12** | **Mean (SD) at T12** | ***p*-value (T0 vs T12)** |
| --- | --- | --- | --- |
| miR-1-3p | -0.172 (-0.965 – 0.807) | -0.027 (1.086) | 0.939 |
| miR-133a-3p | -0.047 (-1.403 – 0.451) | -0.111 (0.864) | 0.694 |
| miR-133b | -0.309 (-1.066 – 0.599) | -0.187 (0.892) | 0.523 |
| miR-206 | 0.864 (-1.547 – 3.245) | 1.089 (2.936) | 0.271 |
| *PGD* [1] | 0.047 (-0.147 – 0.345) | 0.063 (0.393) | 0.645 |
| *G6PD* [1] | 0.008 (-0.215 – 0.332) | 0.026 (0.466) | 0.871 |
| *TKT* [1] | -0.037 (-0.144 – 0.274) | -0.040 (0.371) | 0.755 |
| *HDAC4* [1] | 0.058 (-0.267 – 0.365) | 0.055 (0.309) | 0.996 |
| *FGFR1* [1] | -0.290 (-0.486 – 0.108) | -0.206 (0.438) | 0.196 |
| *SP1* [1] | 0.069 (-0.063 – 0.449) | 0.162 (0.447) | 0.309 |
| *TGFB1* [1] | 0.122 (-0.345 – 0.292) | 0.021 (0.338) | 0.855 |
| *KCNQ1* [1] | -0.111 (-0.286 – 0.362) | 0.018 (0.358) | 0.882 |
| *IGF1R* [1] | 0.191 (-0.594 – 0.411) | 0.037 (0.678) | 0.873 |
| *ANXA2* [1] | 0.225 (-0.017 – 0.307) | 0.180 (0.335) | 0.144 |
| *LINCMD1* [4] | 0.638 (-5.539 – 3.077) | -0.250 (3.817) | 0.879 |
| *lnc-GJA1-2* [2] | 0.009 (-0.460 – 0.380) | -0.161 (1.218) | 0.719 |
| *SMN*-total [1] | 0.197 (-0.333 – 0.539) | 0.156 (0.593) | 0.452 |
| *SMN*-FL [1] | 0.839 (0.338 – 1.329) | 0.751 (0.751) | **0.017** |
| *SMN*-Δ7 [1] | -0.606 (-0.832 – 0.375) | -0.272 (0.811) | 0.515* |
| *SMN*-FL/*SMN*-Δ7 [1] | 1.457 (0.618 – 1.707) | 1.023 (1.230) | 0.086* |

The number of missing data is indicated in [] brackets. Bold values in the table denote statistically significant differences. One-sample t-test was used for normally distributed data, or the Wilcoxon signed-rank test* for non-normally distributed data. Time points T12 for each patient were normalized to baseline samples (T0) and obtained using the 2^-ΔΔCt^ method. SD, standard deviation; IQR, interquartile range.

**Table S15**. Correlation analysis of miRNAs and corresponding target mRNAs, lncRNAs, and *SMN* transcripts (n = 50).

| **RNA interactor** | **miR-1-3p** | | **miR-133a-3p** | | **miRNA-133b** | | **miRNA-206** | |
| --- | --- | --- | --- | --- | --- | --- | --- | --- |
|  | **r** | ***p*-value** | **r** | ***p*-value** | **r** | ***p*-value** | **r** | ***p*-value** |
| miR-133a-3p | 0.309 | **0.029*** | / | / | / | / | / | / |
| miR-133b | 0.425 | **0.002*** | 0.755 | **< 0.001** | / | / | / | / |
| miR-206 | -0.026 | 0.860* | -0.197 | 0.170* | -0.175 | 0.225* | / | / |
| *PGD* [1] | 0.222 | 0.125* | / | / | / | / | -0.278 | 0.054* |
| *G6PD* [1] | 0.047 | 0.751* | / | / | / | / | -0.148 | 0.311* |
| *TKT* [1] | 0.220 | 0.130* | / | / | / | / | -0.158 | 0.278* |
| *HDAC4* [1] | 0.256 | 0.076* | / | / | / | / | -0.350 | **0.014*** |
| *FGFR1* [1] | / | / | 0.342 | **0.016** | 0.405 | **0.004** | / | / |
| *SP1* [1] | / | / | -0.018 | 0.900 | -0.116 | 0.426 | / | / |
| *TGFB1* [1] | / | / | 0.089 | 0.545 | -0.032 | 0.828 | / | / |
| *KCNQ1* [1] | 0.267 | 0.064* | 0.068 | 0.640 | 0.238 | 0.100 | / | / |
| *IGF1R* [1] | / | / | 0.073 | 0.616 | / | / | -0.103 | 0.481* |
| *ANXA2* [1] | -0.061 | 0.677* | -0.047 | 0.749 | 0.071 | 0.626 | -0.065 | 0.657* |
| *LINCMD1* [2] | / | / | -0.195 | 0.184 | 0.105 | 0.477 | / | / |
| *lnc-GJA1-2* [2] | 0.324 | **0.025*** | / | / | / | / | -0.092 | 0.534* |
| *SMN*-total [1] | 0.143 | 0.326* | 0.049 | 0.736* | 0.175 | 0.229* | 0.361 | **0.011*** |
| *SMN*-FL [1] | 0.013 | 0.928* | -0.078 | 0.596 | 0.127 | 0.384 | 0.368 | **0.009*** |
| *SMN*-Δ7 [1] | -0.128 | 0.381* | -0.177 | 0.224 | -0.009 | 0.953 | 0.494 | **<0.001*** |

The number of missing data is indicated in [] brackets. Bold values in the table denote statistically significant differences. Pearson correlation was used for normally distributed data, or Spearman correlation* for non-normally distributed data (Shapiro-Wilk test). r, correlation coefficient.
